# Beringian sub-refugia revealed in blackfish (*Dallia*): implications for understanding the effects of Pleistocene glaciations on Beringian taxa and other Arctic aquatic fauna

**DOI:** 10.1186/s12862-015-0413-2

**Published:** 2015-07-19

**Authors:** Matthew A Campbell, Naoki Takebayashi, J. Andrés López

**Affiliations:** Department of Biology and Wildlife, University of Alaska Fairbanks, Fairbanks, AK 99775 USA; Institute of Arctic Biology, University of Alaska Fairbanks, Fairbanks, AK 99775 USA; School of Fisheries and Ocean Sciences, University of Alaska Fairbanks, Fairbanks, AK 99775 USA; University of Alaska Museum, Fairbanks, AK 99775 USA; Institute of Fundamental Sciences, Massey University, Palmerston North, 4442 New Zealand

**Keywords:** Esocidae, Alaska blackfish, Bering Land Bridge, Isolation with Migration, Glacial Refugia, Range Contraction

## Abstract

**Background:**

Pleistocene climatic instability had profound and diverse effects on the distribution and abundance of Arctic organisms revealed by variation in phylogeographic patterns documented in extant Arctic populations. To better understand the effects of geography and paleoclimate on Beringian freshwater fishes, we examined genetic variability in the genus *Dallia* (blackfish: Esociformes: Esocidae). The genus *Dallia* groups between one and three nominal species of small, cold- and hypoxia-tolerant freshwater fishes restricted entirely in distribution to Beringia from the Yukon River basin near Fairbanks, Alaska westward including the Kuskokwim River basin and low-lying areas of Western Alaska to the Amguema River on the north side of the Chukotka Peninsula and Mechigmen Bay on the south side of the Chukotka Peninsula. The genus has a non-continuous distribution divided by the Bering Strait and the Brooks Range. We examined the distribution of genetic variation across this range to determine the number and location of potential sub-refugia within the greater Beringian refugium as well as the roles of the Bering land bridge, Brooks Range, and large rivers within Beringia in shaping the current distribution of populations of *Dallia*. Our analyses were based on DNA sequence data from two nuclear gene introns (S7 and RAG1) and two mitochondrial genome fragments from nineteen sampling locations. These data were examined under genetic clustering and coalescent frameworks to identify sub-refugia within the greater Beringia refugium and to infer the demographic history of different populations of *Dallia*.

**Results:**

We identified up to five distinct genetic clusters of *Dallia*. Four of these genetic clusters are present in Alaska: (1) Arctic Coastal Plain genetic cluster found north of the Brooks Range, (2) interior Alaska genetic cluster placed in upstream locations in the Kuskokwim and Yukon river basins, (3) a genetic cluster found on the Seward Peninsula, and (4) a coastal Alaska genetic cluster encompassing downstream Kuskokwim River and Yukon River basin sample locations and samples from Southwest Alaska not in either of these drainages. The Chukotka samples are assigned to their own genetic cluster (5) similar to the coastal Alaska genetic cluster. The clustering and ordination analyses implemented in Discriminant Analysis of Principal Components (DAPC) and STRUCTURE showed mostly concordant groupings and a high degree of differentiation among groups. The groups of sampling locations identified as genetic clusters correspond to geographic areas divided by likely biogeographic barriers including the Brooks Range and the Bering Strait. Estimates of sequence diversity (*θ*) are highest in the Yukon River and Kuskokwim River drainages near the Bering Sea. We also infer asymmetric migration rates between genetic clusters. The isolation of *Dallia* on the Arctic Coastal Plain of Alaska is associated with very low estimated migration rates between the coastal Alaska genetic cluster and the Arctic Coastal Plain genetic cluster.

**Conclusions:**

Our results support a scenario with multiple aquatic sub-refugia in Beringia during the Pleistocene and the preservation of that structure in extant populations of *Dallia*. An inferred historical presence of *Dallia* across the Bering land bridge explains the similarities in the genetic composition of *Dallia* in West Beringia and western coastal Alaska. In contrast, historic and contemporary isolation across the Brooks Range shaped the distinctiveness of present day Arctic Coastal Plain *Dallia*. Overall this study uncovered a high degree of genetic structuring among populations of *Dallia* supporting the idea of multiple Beringian sub-refugia during the Pleistocene and which appears to be maintained to the present due to the strictly freshwater nature and low dispersal ability of this genus.

**Electronic supplementary material:**

The online version of this article (doi:10.1186/s12862-015-0413-2) contains supplementary material, which is available to authorized users.

## Background

**Fig. 1:**
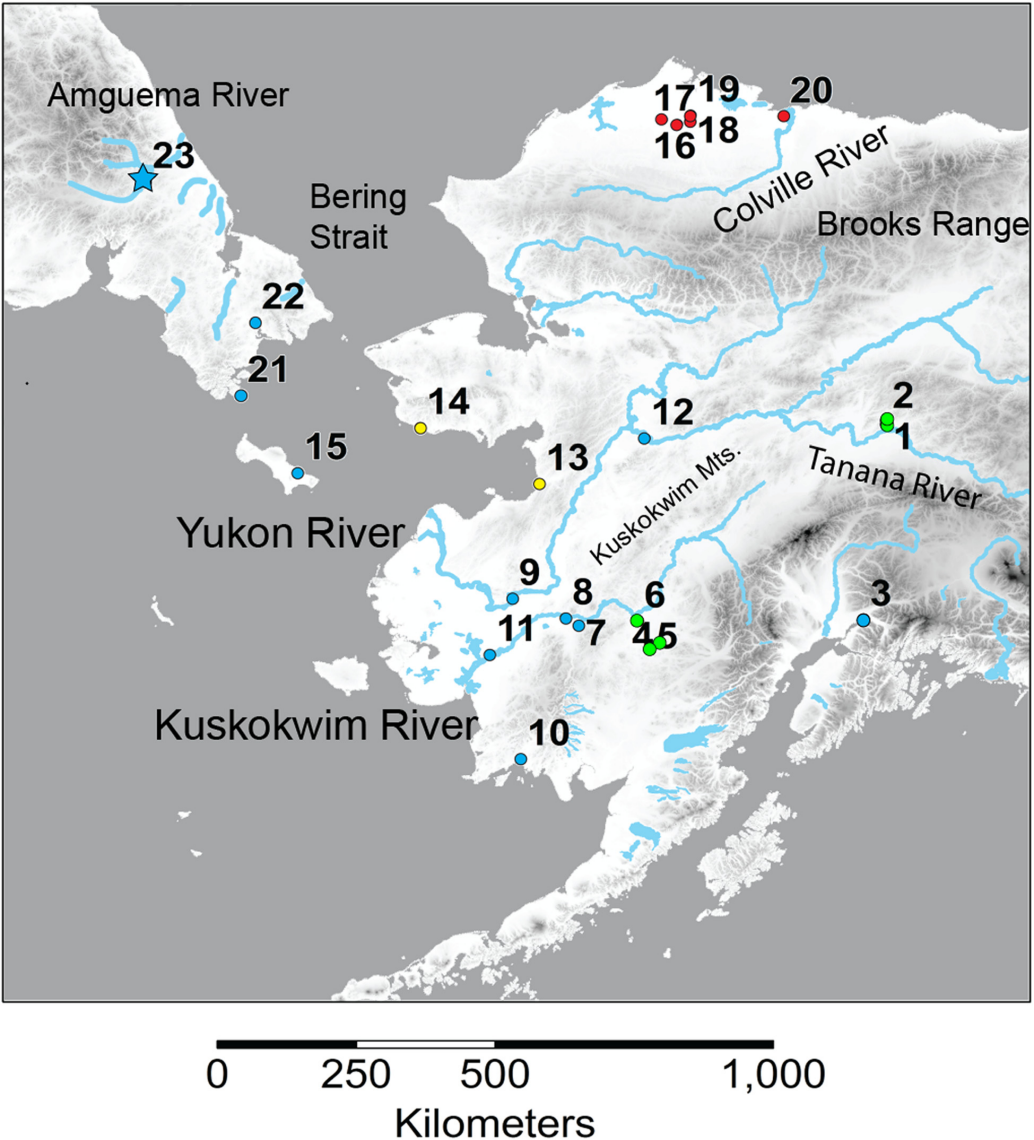
Sampling locations of *Dallia* in Beringia. Distribution of sampling locations of *Dallia* in this study from Alaska, USA and Chukotka, Russia adapted from [19]. Details of collections including number of fish, species, and geographic coordinates are available in Table 1. Major geographic features are labelled that are referred to in the manuscript. Sample locations are indicated by a circle or a star and are color coded to the major mitochondrial lineages identified in [19] as red, blue, green, or yellow. Sample location 23 indicated with a star represents *Dallia admirabilis*, the remainder of sample locations are from *D. pectoralis*. Sample location 3 represents fish from an introduced population of *D. pectoralis*. The physical map contains portions of Alaska and Russia from the Global 30 Arc-Second Elevation (GTOPO30) Digital Elevation Model. Hydrologic features in Alaska are represented through hydrography layers from the Alaska State Geo-Spatial Data Clearinghouse. Major rivers in Chukotka were added to the map by hand

Climatic variation during the Pleistocene strongly influenced the distribution, composition, and genetic diversity of arctic organisms [1]. One method used to understand the effects of paleoclimatic instability on the distribution and evolution of organisms is phylogeography. Phylogeography examines the geographic distribution of genetic variation of organisms to elucidate the role of past and current processes in shaping the distribution of present biodiversity [2]. To date, Holarctic phylogeography studies have relied primarily on the use of mitochondrial DNA (mtDNA) to investigate the effects of paleoclimatic instability, with mammals (*e.g*. [3–9]) receiving most of the attention [10, 11]. Studies of phylogeography have identified the biogeographic region known as Beringia as an important glacial refugium for organisms during the Pleistocene glaciations for a variety of taxa [10]: fishes [12–19], birds [20–23], mammals [3–9, 24–29], plants [30–36], an insect [37], and a lichen [38]. Beringia, with a unique assemblage of flora and fauna, occupies the area adjacent to the former Bering land bridge in both North America and Eurasia [39] (the approximate extent of Beringia is depicted in Fig. 1).

The role of Beringia as an aquatic refugium has been documented in several freshwater fish species [12–19]; however, three points are worth highlighting regarding Holarctic fish phylogeography. First, the amount of phylogeographic concordance among fishes from glaciated areas is low [40]. That is, due to differences in histories of survival and dispersal of genotypes, phylogeographic patterns differ among species in the region. Second, Holarctic phylogeographic studies to date have not included intensive sampling within Beringia. Sampling efforts in many Holarctic phylogeography studies have focused on obtaining samples across the entire range of target species, which typically is very large in the case of Holarctic freshwater fish species. Beringia may be a small component of an organism’s range or peripheral to the main distribution of the organism, and therefore is regularly represented by few sampling sites and a low number of individuals. Finally, studies of Holarctic freshwater fish phylogeography initially relied nearly entirely on mitochondrial data. While mtDNA offers clear practical advantages in studies of this type [41], it is maternally inherited as a single non-recombining locus and therefore provides a very limited window into the genealogical histories of populations [42]. Investigations are now incorporating additional DNA markers to overcome limitations of mtDNA only datasets. It appears that the only emergent fact from diverse studies of freshwater fish phylogeography around and in Beringia is that the Beringian refugium was present, but the geographic extent of this refugium for aquatic organisms and the potential subdivisions of the Beringian refugium remain largely unknown; however, sub-refugia have been identified previously within Beringia [*e.g*. 10, 18].

Our understanding of glacial refugia is of interest as conditions occurring around refugia generate situations considered conducive to speciation and rapid evolution. As glaciers extended across the landscape, freshwater habitats became increasingly circumscribed. As a result, glaciations stressed freshwater organisms with both environmental extremes (strong selective pressures) and further subdivided freshwater fish species (decreasing gene flow), decreasing population sizes (increasing genetic drift). With glacial retreat, isolated populations may reconnect leading to gene flow between differentiated gene pools. Alternatively, populations may remain isolated and continue on the path to allopatric speciation. Uncovering signatures of refugia and sub-refugia is also of interest since inferred refugia discovered through the study of one species may prove to have served that same role for other organisms with similar habitat requirements. To extend our knowledge of Beringian sub-refugia and the impact of Pleistocene climatic instability on freshwater organisms, we studied the phylogeography of the genus *Dallia* (Blackfish; Esociformes: Esocidae [43]).

All extant populations of *Dallia* are confined to Beringia [44], a unique trait among strictly freshwater fishes. Furthermore, also unique among Beringian fishes *Dallia* can obtain atmospheric oxygen through air breathing [45]. *Dallia* are small fish, and rarely exceed 20 cm in length though they may grow in excess of 30 cm [46, 47]. Individuals have been documented to reach eight years of age, but fish in the 0-5 age range comprise the majority of individuals in studied populations [48–50]. Overall the fish is roughly cylindrical in body shape with rounded fins. The dorsal and anal fins are far back along the body and nearly opposite. The caudal fin is not used as a primary locomotive source in these species, instead the large pectoral fins provide most of the propulsion.

Throughout their range, *Dallia* prefer slow-moving or still waters that are heavily vegetated, which may be glacial lakes with small gravel substrate, muddy bottomed thermokarst lakes, or slow moving streams. On the Chukchi Peninsula, *Dallia* are most abundant in shallow thermokarst lakes that are silt bottomed and overgrown with plants [48]. In Alaska, *Dallia* are known to inhabit interior Alaskan streams, lakes in the Bristol Bay region, and tundra lakes and polygon ditches on the Arctic Coastal Plain [49–51].

The ecology and physiology of *Dallia* have not been intensely studied [45, 48–52]. But there is evidence of extensive phenotypic variability between populations. In particular, the growth rate and size at maturity varies greatly among habitats and locations. Observed rates of growth in interior Alaska are much higher than those in Southwest Alaska in the Bristol Bay area [49, 50]. Blackett [50] also notes that females were mature at 80 mm, at age 1 or 2. In contrast, individuals from Lake Aleknagik reach maturity at lengths between 49 and 50 mm and age 3 [49]. Morphology, inferred locomotive capability, and habitat preference (see above) and details of the migratory behaviour of *Dallia* strongly support the speculation that long-distance migrations do not occur in this species [49, 50].

The range of *Dallia* when compared to other Holarctic fishes is comparatively small. In Russia, populations are found on the northeastern edge of the Chukotka Peninsula from the Amguema River in the north eastward along the coast where several species have been described [53, 54], to Lake Achchen in the vicinity of Mechigmen Bay [48]. The distribution of *Dallia* in Alaska includes a central portion of the Arctic Coastal Plain of Alaska, the Yukon River Basin, the Western and Southwestern Alaskan coastal plain, and some Bering Sea Islands between Alaska and Russia [47, 50, 55]. The Brooks Range and the Chukchi Sea separate the populations found on the Arctic Coastal Plain of Alaska from all others (Additional file 5: Figure S1).

Because these species are characterized by limited seasonal movements and short lifespans, we earlier predicted a higher degree of genetic spatial structuring in *Dallia* when compared to other Beringian fishes [19]. An analysis of patterns of variation and distribution of mtDNA sequences [19] revealed several potential sub-refugia within the greater Beringia refugium [56] and a higher degree of mitochondrial variability in comparison to fishes with comparable latitudinal distributions. In addition, the hypothesis that glacial cycling led to isolated species of *Dallia* in Asia [53, 54] was not supported by mtDNA evidence, rather a core of *Dallia* mitochondrial diversity was found in Southwest Alaska. Interestingly, populations from regions adjacent to the former Bering land bridge are very similar. Isolation within Alaska is clear, in particular fish from the Arctic Coastal Plain were found to be isolated both in terms of a disjunct distribution but possessing a unique mitochondrial lineage. Areas which are connected by major river systems, the Yukon and Kuskokwim rivers, did not show mixing of haplotypes except in a single individual, and in general distinct biogeographic areas hosted diagnostic mitochondrial lineages. However, the history of mtDNA lineages represents a single sample of genetic lineage histories in the evolution of an organism. Results from mtDNA data alone are limited, and particular hazards exist by overlooking the nuclear genome such as observing only patterns reflecting processes affecting females or direct or indirect selection on mtDNA producing undependable results.

To better understand the effects of paleoclimate on Beringian freshwater fauna, and to provide a more complete genetic perspective on relationships of *Dallia*, we examined combined mitochondrial and nuclear genetic variability in *Dallia*. We examined DNA sequence variation at mitochondrial and nuclear loci from specimens sampled from across the geographic range of the genus in Alaska, and from three locations in eastern Russia including the type locality of *D. admirabilis*. We investigated the demographic history of *Dallia* with multilocus coalescent methods to estimate genealogical relationships between and within Russian and Alaskan populations of *Dallia*. In this study we ask the following questions: 1) What are the genealogical relationships among sampled populations of *Dallia*? 2) Do nuclear and mtDNA data provide concordant results? 3) How many Beringian glacial sub-refugia are compatible with observed levels and distribution of genetic variability in extant populations of *Dallia*? 4) How did the Bering land bridge affect distribution and movements of ancestral populations of *Dallia*? Additionally, we provide a genetic perspective on the taxonomic status of different *Dallia* populations within our study.

## Methods

### Sample collection

We sampled *Dallia* from localities throughout its geographic range on the Alaska mainland, Saint Lawrence Island, and from three locations on the Chukotka Peninsula (Fig. 1, Table 1). The sample set included collections of *D. pectoralis* from the Arctic Coastal Plain of Alaska and fish from the type locality of *D. admirabilis* in Russia to further refine taxonomic questions in this genus. A total of 188 individuals collected between October 2008 and June 2010 were utilized in this study. We also examined samples of Russian *D. pectoralis* and *D. admirabilis* from Siberia from the fish collection at the Burke Museum of Natural History and Culture, Seattle, Washington, USA (catalog numbers: UW 041669, UW 041670, UW 041671). The samples examined include fishes used in a karyotype and morphology study of *D. pectoralis* in Alaska [57] and were received from the Royal Ontario Museum, Toronto, Canada (accessions 5722 and 5844). DNA was extracted from fin clips if available, but also gill arches and muscle tissue using Qiagen DNEasy spin-column kits. The procedure is described in [19].

**Table 1:** Sampled locations geographic placement, geographic coordinates, sample size and species of *Dallia* sampled

Location Number	Place	Latitude	Longitude	N	Putative Species
1	Fairbanks	64.8692	−147.8254	10	*D. pectoralis*
2	Fairbanks	64.9117	−147.8288	10	*D. pectoralis*
3	Wasilla	61.5374	−149.2550	5	*D. pectoralis*
4	Kuskokwim Basin	61.1945	−156.1535	4	*D. pectoralis*
5	Kuskokwim Basin	61.0812	−156.4840	9	*D. pectoralis*
6	Kuskokwim Basin	61.5597	−156.9341	1	*D. pectoralis*
7	Kuskokwim Basin	61.4300	−158.9114	2	*D. pectoralis*
8	Kuskokwim Basin	61.5406	−159.3765	1	*D. pectoralis*
9	Russian Mission	61.7952	−161.2443	15	*D. pectoralis*
10	Togiak	59.0546	−160.3962	12	*D. pectoralis*
11	Bethel	60.7904	−161.7799	16	*D. pectoralis*
12*	Galena	64.7167	−157.0000	12	*D. pectoralis*
13	Unalakleet	63.8099	−160.7590	11	*D. pectoralis*
14	Nome	64.5061	−165.4305	15	*D. pectoralis*
15	St. Lawrence Island	63.3451	−169.4893	5	*D. pectoralis*
16	Arctic Coastal Plain	70.2768	−156.9182	6	*D. pectoralis*
17	Arctic Coastal Plain	70.1981	−156.1973	2	*D. pectoralis*
18	Arctic Coastal Plain	70.2528	−155.5849	3	*D. pectoralis*
19	Arctic Coastal Plain	70.3683	−155.5697	4	*D. pectoralis*
20*	Colville River	70.3333	−151.2000	6	*D. pectoralis*
21+	Novoe Chaplino	64.4085	−172.2590	10	*D. pectoralis*
22+	Ievineem River	65.6808	−172.5542	10	*D. pectoralis*
23+	Amguema Basin	67.4346	−178.6985	8	*D. admirabilis*

**Fig. 2:**
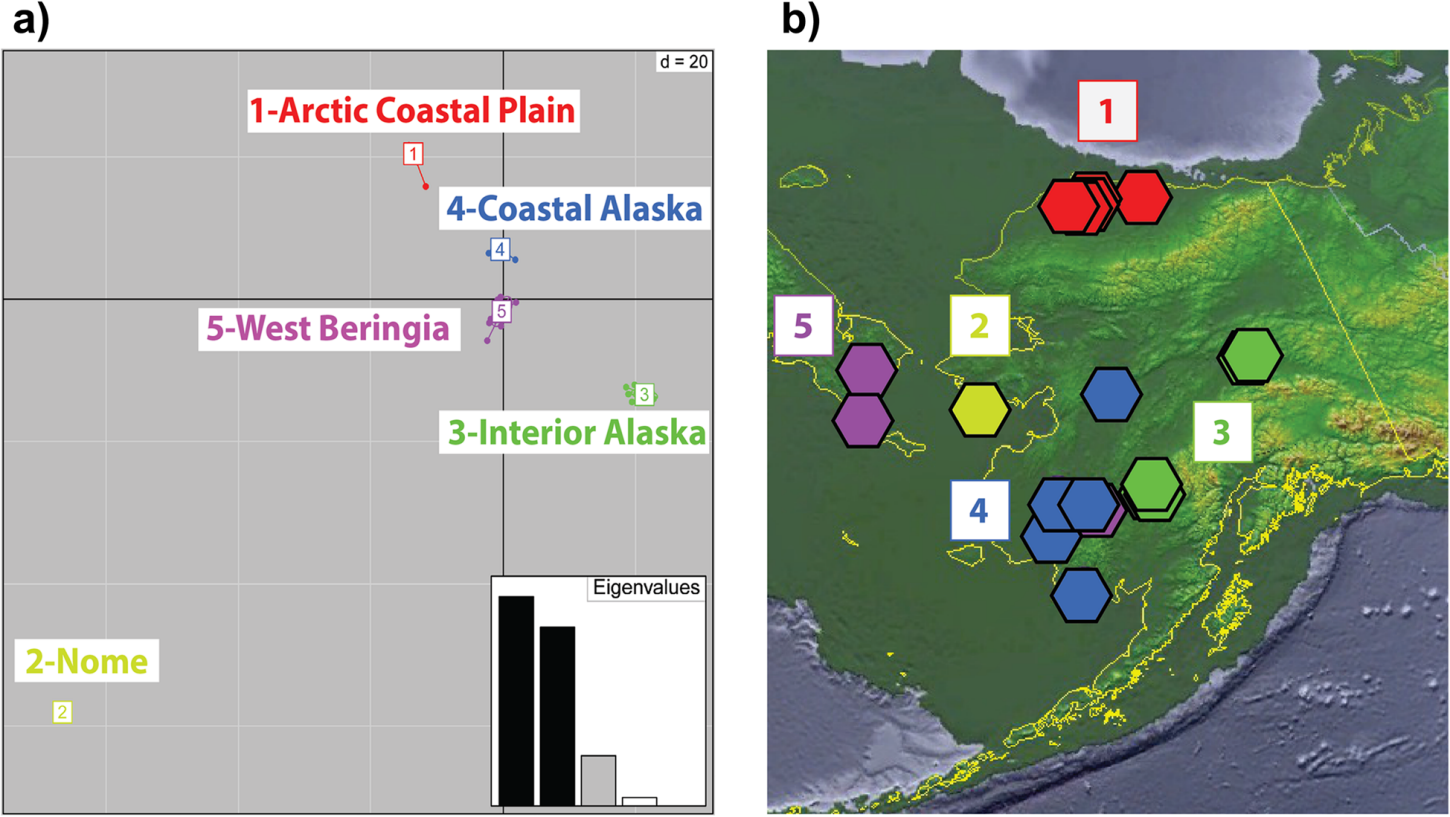
DAPC scatterplot for *K* = 5 and geographic distribution of genetic clusters. **a**) DAPC scatterplot for *K* = 5, depicting the clustering of individuals based on K-means clustering of principal components. Eigenvalues representing between to within group variation for linear combinations of principal components are included in this figure. Cluster 1 “Arctic Coastal Plain” (locations 16-20 [n = 19]); Cluster 2 “Nome” (location 14 [n = 3]); Cluster 3 “interior Alaska” (locations 1-2, 4-6 [n = 21] and 12 [n = 1]); Cluster 4 “Coastal Alaska” (locations 8-12 [n = 17]); and Cluster 5 “West Beringia” (locations 21-22 [n = 19], 7 [n = 2], and 11 [n = 1]). **0062**) The sampling locations color-coded to the corresponding clusters in part a), shown on a map of Beringia. Arctic Coastal Plain (red), Nome (yellow), interior Alaska (green), coastal Alaska (blue), and West Beringia (purple). The map includes a -100 meter sea level (dark green layer) corresponding to the last glacial maximum available from the US National Geophysical Data Center

Adapted from [19]. Sample location numbers which correspond to Fig. 2, geographic place name where the sample was collected, coordinates (WGS 84) of sample, and sample size (N) of *Dallia* obtained for this study in 2008-2010, from Alaska, USA and Chukotka, Russia. Samples from the karyotype study of Crossman and Rab [57] are indicated by an asterisk (*), Burke Museum collections are indicated with a plus sign (+)

### Sequence data

Our dataset comprises DNA sequences from portions of the following four gene regions: intron 2 of the recombination activating gene I (RAG1-I2), intron 1 of the S7 ribosomal protein (S7-I1), and the mitochondrial control region (CR) and cytochrome oxidase I gene (COI). The first two genes are located in the nuclear genome.

Primers for amplification and sequencing of RAG1-I2 bind to the exons flanking this intron and are specific to esociform fishes [58]. We compared sequenced products to known esociform RAG1-I2 sequences on GenBank with the basic local alignment search tool (BLAST) to verify amplification of the correct genomic region. Amplification conditions for RAG1-I2 were 1X ProMega GoTaq Flexi reaction buffer, 0.2 mM dNTP's, 2 mM MgCl_2_, 0.4 μM forward primer, 0.4 μM reverse primer, 0.025U/μL GoTaq Flexi Taq polymerase, and 1 μL template of variable concentrations. For this primer set, the thermocycler was programmed to an initial denaturing of 94 °C for 2 min, followed by 30 cycles of denaturing at 94 °C for 20 s, annealing at 60 °C for 25 s, and 72 °C for 50 s with a final extension step at 72 °C for 2 min. Several degraded samples required us to increase the number of PCR cycles to 40 to generate sufficient product for sequencing.

Universal fish primers targeting S7-I1intron were used for amplification and sequencing [59]. We confirmed specificity of the amplification products by the similarity between our S7-I1 sequences and those currently available on GenBank. PCR reagent concentrations for S7-I1 were the same as those used for RAG1 in this study. The thermocycler profile was the following: An initial denaturing at 95 °C for 2 min followed by 35 cycles and a final extension. Each cycle comprised a 95 °C denaturing for 30 s, 55 °C annealing step for 1 min, and 72 °C extension step for 1 min and 30 s. A final 2 min extension step at 72 °C ended the profile. For both introns, unincorporated primers and dNTPs were removed enzymatically using the ExoSAP-IT protocol. Purified PCR products were sequenced using ABI Big-Dye v3.1 chemistry on ABI 3730XL automated sequencers. PCR product purification and sequence determination were performed by High-Throughput Sequencing Solutions at the University of Washington, Seattle, U.S.A.

The mitochondrial CR and COI sequences examined have been previously described [GenBank JX961713–JX962051] [19]. These fragments were concatenated for each individual because the mitochondrial genome is non-recombining. All newly determined DNA sequences examined in this study are available in GenBank under accession numbers KP411389 - KP411458 and KP411459 - KP411534.

Sequences were aligned using CodonCode Aligner version 3.0.3 [60] and haplotypes of nuclear alleles were determined using PHASE version 2.1 [61, 62] as implemented in DnaSP version 5.10 [63]. The latter was also used to estimate basic summary statistics of genetic diversity such as alignment length, the number of variable sites, haplotype diversity (HD), average pairwise differences (k), nucleotide diversity (π) and tests of neutrality (Fu and Li’s D, Fu and Li’s F, and Tajima’s D), and to test for evidence of recombination with the four-gamete test [63, 64].

### Determination of population structure

We used two different methods for comparative purposes. For both of these genetic clustering algorithms we utilized individuals from which at least one nuclear intron sequence was available.

The first method to evaluate population structure was Discriminant Analysis of Principal Components (DAPC) from the *adegenet* R package [65, 66]. Unlike the STRUCTURE method, DAPC is not based on an underlying population genetics model. DAPC accounts for arbitrary linkage structures among single nucleotide polymorphic sites by transforming observed polymorphisms into principal components [67]. The conversion from genetic data into principal components permits the use of generic clustering techniques such as K-means clustering and discriminant analysis. For DAPC, we converted our sequence data to single nucleotide polymorphisms (SNPs). The SNP data were imported into R as separate diploid and haploid (mtDNA) data and then combined with the *adegenet* package [65]. To perform DAPC we identifed population clusters with the *find.clusters* function within *adegenet*. For each *k* of population clusters, *find.clusters* calculates the Bayesian information criterion (BIC) for the corresponding K-means for each *k*. The resulting plot of BIC scores for K-means of each *k*, aids identification of the most appropriate number of *k* groups by identifying the appropriate amount of parameterization for the data. The population clusters identified by *find.clusters* were used as prior assignments in DAPC [65, 67]. DAPC first performs Principal Component Analysis (PCA, *dudi.pca*) and Discriminant Analysis (DA, *Ida*) utilizing the packages *ade4* [68–70] and *mass* [71].

Our second approach to identify genetic clusters, was STRUCTURE version 2.3.3 [72, 73]. Unique mitochondrial haplotypes and nuclear alleles were coded as numbers and entered into STRUCTURE version 2.3.3 [72]. Using a burn-in of 100,000 steps followed by a Markov Chain Monte Carlo (MCMC) process of 200,000 steps, the likelihood of number of genetic clusters (*K*) within *Dallia* were calculated for *K* = {1,…,8} in two independent runs. In STRUCTURE, we specified the admixture and correlated allele frequency ancestry and frequency models respectively. For each value of *K* we allowed the alpha parameter to be inferred. The two independent structure runs for each *K* were compared to each other to assess convergence and averaged for comparison to other values of *K*. For *K* = {1,…,8} the negative natural log likelihood of the probability of data given a particular *K* (-lnP (X | *K*)) was compared across *K* values to identify the optimum *K*. The value of -lnP (X | *K*) increased towards *K* = 4 then declined, indicating an optimum. Visualization of the output from STRUCTURE was done with Distruct [74].

### Do nuclear data support genetic clusters of *Dallia*?

As we expected much more variation in mitochondrial data, it was important to establish that the nuclear data provided some information regarding genetic relationships among sampled locations of *Dallia*. Secondarily, we investigated nuclear signal to examine congruency between the two data types. We applied DAPC to the nuclear intron dataset alone to determine if and what genetic clusters are supported by the nuclear component of our dataset.

### Demography and pattern of gene flow

Pairwise comparisons between genetic clusters of *Dallia* were performed in Isolation with Migration (IM) [75, 76] to estimate the patterns of connectivity among the genetic clusters (populations) identified in the analyses described above. While clustering results from DAPC and STRUCTURE were highly congruent, we chose the finer partition results from DAPC in the IM analysis. Of the five clusters defined by DAPC, only four had sufficient membership for IM analyses. Individuals excluded from the population structure analyses because they were represented only by mitochondrial sequence data were included in IM analyses as appropriate. Salient parameters of demographic history, such as population size, divergence time and migration rates can be estimated in an IM analysis. Interpretation of IM results permits the evaluation of the effects of biogeographic barriers such as the Bering Sea by comparing migration rates between populations on either side of this barrier. To identify major patterns of migration, separation, and genetic diversity across the range of *Dallia*, we used a series of pairwise IM comparisons.

IM uses a MCMC algorithm to simulate genealogies based on a two-population and six-parameter model. We used IM to estimate the divergence time (*t*) at some time in the past for two populations. Migration rates between the two descendent populations were estimated (*m*_1_ and *m*_2_). The population genetics parameter *θ*, which combines effects of effective population size (N_E_) and mutation rate (μ),was estimated individually for three populations in each IM analysis: the ancestral population, and both descendent populations (*θ*_A_, *θ*_1_, and *θ*_2_.). We did not choose to estimate the change in population size (*s* parameter) in these IM analyses. Importantly, each parameter in IM is combined with mutation rate (μ) such that *t* = t (generations) * μ *m*_1_ = m (individuals/generation)/μ, *θ*_1_ = 4*N_1_ (effective population size 1) * μ.

Each pairwise comparison between *Dallia* populations consisted of several IM analyses. Initially, prior distributions with wide ranges were specified for the six parameters to determine suitable upper limits for priors in subsequent analyses. Several iterations of assessing convergence and constraining priors were conducted. Final convergence was evaluated by repeated long MCMC searches with differing seed numbers and comparing results.

## Results

### DNA sequencing

The final dataset includes sequences of RAG1-I2 from 70 individuals, of S7-I1from 77 individuals, and mtDNA data [19] from 124 individuals at both mitochondrial loci (Table 2). These samples represent 19 collecting localities. Samples from locations 13, 15, and 23 did not yield sequences from the nuclear loci. We also collected data from what is presumed to be an introduced population of *D. pectoralis* (sample location 3), but omitted those data from the clustering and demographic analyses presented in this study.

**Table 2:** Summary of alignment length, polymorphism, and tests of neutrality for the mtDNA, RAG1-I2 and S7-I1 alignments used in this study

Alignment	Number of Sequences	Alignment Length (base pairs)	Variable Sites	Number of Haplotypes	Haplotype Diversity (HD)
**mtDNA**	**124**	**1230**	**64**	**35**	**0.94**
**RAG1 I2**	**140**	**730**	**9**	**8**	**0.52**
**S7 1**	**154**	**750**	**10**	**10**	**0.50**
	**Average Pairwise Differences** **(k)**	**Nucleotide Diversity** **(π)**	**Fu and Li'** **s D**	**Fu and Li'** **s F**	**Tajima'** **s D**
**mtDNA**	**10.31**	**0.0084**	**0.59**	**0.62**	**0.42**
**RAG1 I2**	**1.80**	**0.0025**	-**1.61**	-**1.24**	-**0.02**
**S7 1**	**0.58**	**0.00077**	-3.16*	-3.11**	-**1.62**

Descriptions of each of the three alignments in this study, including number of sequences, length, basic polymorphism data, and tests of neutrality. Significance of neutrality tests is indicated by an *(P < 0.05) or **(P < 0.02)

The haplotypic phase of heterozygous individuals was resolved with high posterior probabilities (>0.90) for all but three individuals in the RAG1-I2 alignment and another set of three individuals in the S7-I1 intron alignment. We have used the highest probability phase for the following analyses and the robustness of assignment is confirmed by separate iterations of PHASE with different seeds. Ambiguous sites within an individual not having a phase posterior probability > 0.90 are coded as missing data for use in analyses requiring phase information such as IM and recombination test. For calculating summary statistics based on site-frequency spectra (Table 2), all data including unphased sequences are included. DAPC does not require phase info since it is based on SNP genotypes, therefore SNP sites with low posterior probability for phase were included. Testing of recombination under the four-gamete test did not indicate recombining areas within our alignments.

### Determination of population structure

Results from DAPC indicate that there are five separate genetic clusters of sample locations (Fig. 2a and b; Individual assignments are shown in Additional file 1 and comparisons of different *K* are presented in Additional file 2). Cluster 1 consists of fish from the Arctic Coastal Plain (sample locations 16-20). Cluster 2 contains fish from the Nome sample location (14). Cluster 3 is composed of sample locations from the Tanana River and Kuskokwim River upstream of the Kuskokwim Mountains (interior Alaska, sample locations 1, 2, & 4-6), and one individual from the lower Yukon River (sample location 12). Cluster 4 contains western coastal Alaska sample locations including Yukon and Kuskokwim locations downriver from sample locations 1, 2, & 4-6 (coastal Alaska, sample locations 8-12). Cluster 5 is made up of fish from sample locations in Chukotka, Russia (West Beringia, sample locations 21 & 22), but also three individuals from Southwest Alaska (sample locations 7 and 11). Probability of assignment into all groups is very high for all individuals (DAPC output for assignment probabilities can be found in Additional file 1). Over the entire dataset, only four individuals are assigned to groups from a different geographic area from where they were sampled. From sample location 7 (n = 2/2) and sample location 11 (n = 1/6) in Southwest Alaska three fish are found to be clustered with sample locations 21 and 22 from Chukotka (Cluster 5). A single fish from sample location 12 (n = 1/4; all others assigned to Cluster 4 [coastal Alaska]) is assigned to Cluster 3 (interior Alaska). F_st_ estimates were calculated from groups identified in DAPC in a pair-wise fashion, range between 0.32 and 0.91, and are shown in Table 3.

**Table 3:** DAPC estimates of F_st_ based on pair-wise comparisons of genetic clusters

Geographic Area	West Beringia	Interior Alaska	Arctic Coastal Plain	Nome
**Interior Alaska**	0.63	−	−	−
**Arctic Coastal Plain**	0.61	0.38	−	−
**Nome**	0.85	0.67	0.67	−
**Coastal Alaska**	0.49	0.36	0.32	0.91

Pairwise comparisons of F_st_ for genetic clusters identified in the DAPC analysis

**Fig. 3:**
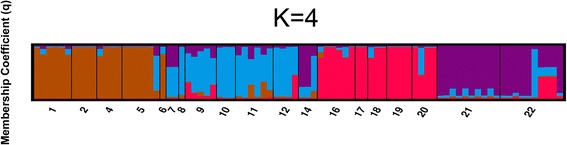
Distruct graph of STRUCTURE for groups of sampled populations (*K*) of four. Distruct graph of STRUCTURE output for assignment of individuals to sampled populations (*K*), of *K* = 4. Each column represents an individual with the amount of color indicating the posterior probability of an individual being assigned to a group of that color (membership coefficient [*q*]). Sampling location number is indicated on the *x* - axis and correspond to Fig. 2

The optimum number of groups of the nineteen sampled localities found by the STRUCTURE algorithm is four (Fig 3, comparisons of different *K* are presented in Additional file 2). The four groups correspond to four geographic areas: 1) the Arctic Coastal Plain of Alaska (sample locations 16-20); 2) the Tanana River drainage and Kuskokwim River drainage upstream of the Kuskokwim Mountains (interior Alaska, sample locations 1, 2, & 4-6); 3) western coastal Alaska and the lower Yukon and Kuskokwim river systems sample locations 7-12 (coastal Alaska); and 4) Russian and Nome sample locations (21, 22, and 14). STRUCTURE provides estimates of fixation index, F_st_, from an ancestral population for each of the four groups of sampled populations identified. For the groups listed above, the F_st_ values in order are: 0.47, 0.68, 0.0024, and 0.44.

### Do nuclear data support genetic clusters of *Dallia*?

DAPC applied to a nuclear intron dataset of eighty-three individuals total with nineteen individuals represented by only one locus supports three genetic clusters (Additional file 3). Samples from the Arctic Coastal Plain were determined to be a separate genetic cluster (n = 18; sample locations 16-20, with one individual from sample location 12) as were samples from interior Alaska (n = 17; sample locations 1, 2, & 4-6). Samples from the remaining natural distribution of *Dallia* including western coastal Alaska, Chukotka, and Nome are all placed into a single remaining cluster (n = 48, sample location 7 [n = 2], sample location 8 [n = 1], sample location 9 [n = 5], sample location 10 [n = 3], sample location 11 [n = 6], sample location 12 [n = 3], sample location 14 [n = 3]; sample location 21 [n = 10], sample location 22 [n = 9]) with some individuals placed in this group from other geographic regions (Arctic Coastal Plain sample location 16 [n = 1] and sample location 20 [n = 1]; interior Alaska sample location 1 [n = 2], sample location 4 [n = 1], sample location 5 [n = 1]).

### Samples included in IM analyses and parameter convergence

Data from 19 sampling locations were grouped into five distinct genetic clusters as indicated by DAPC. We included the following four genetic clusters as populations in IM analyses (Nome was excluded due to small sample size): coastal Alaska, West Beringia, interior Alaska and Arctic Coastal Plain with sample sizes shown in Table 4. We do not have a comprehensive hypothesis of the relationships among these defined populations; however, the core diversity of *Dallia* was clearly visible in the coastal Alaska genetic cluster. We then considered the coastal Alaska population to be the core population of our study, with the three other (West Beringia, Arctic Coastal Plain and interior Alaska) to be peripheral. Under this model we conducted three pairwise comparisons between the Coastal Alaska population and each of the other three populations.

**Table 4:** Number of locations and sequences used for each population in IM analyses

	Number of:				
Population	Sampling Locations	Individuals	mtDNA Sequences	RAGI I2 Sequences	S7 1 Sequences
**Coastal Alaska**	**6**	**58**	**58**	**26**	**36**
**West Beringia**	**2**	**19**	**13**	**28**	**38**
**Interior Alaska**	**5**	**32**	**32**	**40**	**34**
**Arctic Coastal Plain**	**5**	**21**	**21**	**34**	**36**

Each population used for IM analyses in this study and the number of sampling locations that compose each population are listed. Populations were determined by DAPC. The number of sequences of each type of genetic data is also summarized

Parameter convergence is possible for all parameters except *Θ*_A_. For the coastal Alaska and West Beringia and coastal Alaska and Arctic Coastal Plain comparisons a flat posterior probability distribution for *Θ*_A_ is produced. In the coastal Alaska and interior Alaska population comparison, the posterior probability distribution of *Θ*_A_ has a clear peak but the tail of the distribution extends past 200. While the *t* parameter appeared to converge, effective sample size (ESS) values for this parameter remained below 50 between all runs. Output from IM analyses is available as posterior distributions in Additional file 4, and details of parameter distributions including ESS values in Additional file 7: Table S3.

### Migration rates

**Fig. 4:**
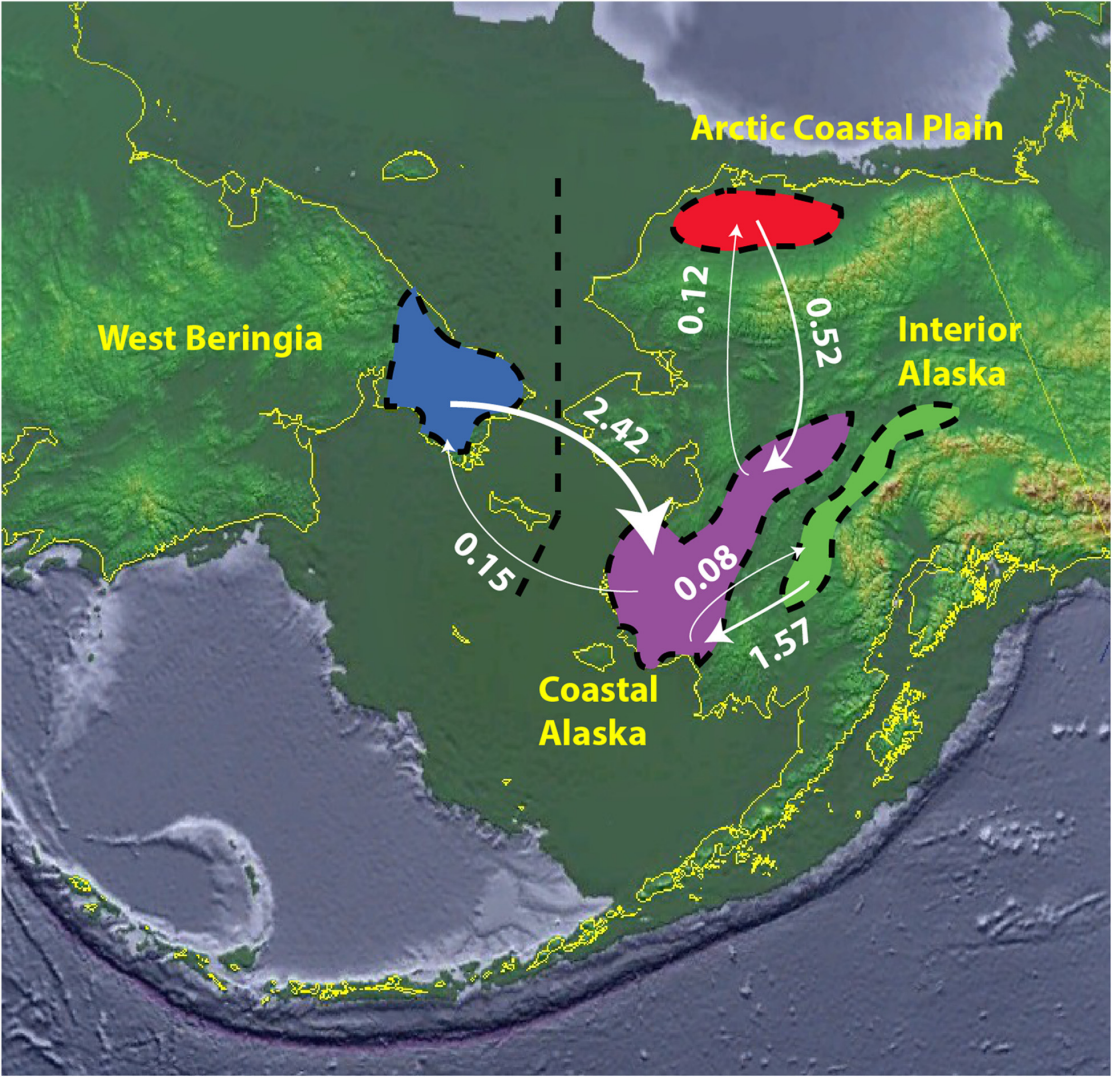
Migration rates among Beringian *Dallia* populations. Migration rates in individuals per generation since the time of divergence (*t*) between populations of *Dallia* estimated by Isolation with Migration analyses. Approximate geographic distribution of populations defined by clustering algorithms are outlined with black dashed lines. Arrows reflect the magnitude of migration rate. Current coastlines and international borders (yellow lines) are overlaid onto a map of -110 meter sea level (dark green layer) corresponding to the last glacial maximum available from the US National Geophysical Data Center

Estimates of migration rates between populations in the IM comparisons were in general asymmetrical and ranged between 0.08 and 2.42 individuals per generation on average since the time of divergence between each population pair (calculated by (mean population *θ* x mean migration rate *m*)/2) (Fig 4). The migration rate from the coastal Alaska population to the other three populations was consistently smaller than the reverse. The smallest overall migration rates are observable in the Arctic Coastal Plain and coastal Alaska comparison. The largest inferred migration rate is from West Beringia to coastal Alaska.

### Estimates of *Θ*

The estimated *θ* for the coastal Alaska population (4.10 to 5.68) is consistently much larger than that for the populations with which it was compared which range from 0.22 to 0.62 (Table 5). There is no overlap in the 90 % highest probability density between the estimate of *θ* for the coastal Alaska population and the other (peripheral) populations.

**Table 5:** Estimates of *Θ* from the three Isolation with Migration comparisons

Comparison	Coastal *θ*	HPD90Lo	HPD90Hi	Other *θ*	HPD90Lo	HPD90Hi
**Coastal Alaska and West Beringia**	5.68	3.04	10.14	0.53	0.15	1.23
**Coastal Alaska and Interior Alaska**	4.10	2.02	7.61	0.62	0.28	1.30
**Coastal Alaska and Arctic Coastal Plain**	4.88	2.88	7.94	0.22	0.05	0.69

Values for the parameter *θ* (4 x effective population size x mutation rate) are given for the three pairwise Isolation with Migration (IM) comparisons. Without a comprehensive hypothesis of relationships between the populations defined by DAPC, IM analyses between the core diversity of the coastal Alaska population and other three defined populations of sufficient sample size (West Beringia, interior Alaska, and the Arctic Coastal Plain) were conducted. Only *θ* for the contemporary populations is shown. The mean of posterior distributions is presented with the 90 percent highest probability densities (HPD90Lo and HPD90Hi). Other *θ* indicates the non-coastal Alaska member of the pairwise comparision

## Discussion

The study was undertaken to evaluate patterns of survival and dispersal within Beringia during the Pleistocene and any role Pleistocene climatic instability may have had in diversification of this genus. Our results have demonstrated unique findings at a higher resolution within Beringia than other studies of freshwater fishes. We provide evidence for sub-structuring within the greater Beringia refugium, and with combined nuclear and mitochondrial data, estimates of migration rates and *Θ* among genetically structured populations are available. The results of the study are important for better understanding the role of Beringia as an aquatic refugium during the climatic instability of the Pleistocene glaciations, the influence of glaciations on freshwater fish genetic diversity, and the role of the Bering land bridge in aquatic migration.

### Sub-Refugia in Beringia

The two genetic clustering algorithms used as population boundary identification methods (DAPC and STRUCTURE) provided evidence of strong population structure across the current geographic range of *Dallia* consistent with biogeographic barriers such as the Bering Strait and mountain ranges. Subdivision of *Dallia* within Beringia into up to five different genetic clusters was supported by DAPC. The results of DAPC and STRUCTURE generally identified divisions among sampling locations in a congruent manner, the exception of the Nome sample location (sample location 14), which the DAPC analysis resolved as a distinct genetic cluster. The lack of support in STRUCTURE for more than four populations is likely a result of haplotype coding instead of SNP coding which was used in DAPC, perhaps resulting in an inability to further resolve population subdivisions. Nonetheless, both STRUCTURE and DAPC consistently supported a North-South Beringia divide in population structure. The Arctic Coastal Plain *Dallia* are genetically distinct from those found south of the Brooks Range, which has also been observed in other Beringian fishes and had been hypothesized for *Dallia* [14, 18, 53, 54, 77]. Significant differentiation between interior Alaska and Southwest Alaska has been demonstrated with microsatellite data from *Dallia*, corroborating further subdivision of the greater Beringian refugium beyond a North-South Beringia divide [78] presented in this manuscript. The five genetic clusters identified in this study correspond to major geographic areas and barriers such as the Brooks Range, Bering Sea, the Kuskokwim Mountains and Yukon/Tanana rivers. In comparison to other studies of Beringian freshwater fishes, we found finer resolution of population structure within the greater Beringia refugium. In Arctic grayling (*Thymallus arcticus*), mitochondrial and microsatellite DNA identified two refugia within Beringia- North and South Beringia though fewer sampling locations were used [18]. The North Beringian Arctic grayling stock perhaps originated North of the Brooks Range perhaps in a parallel history to that of the Arctic Coastal Plain *Dallia* in this study. The south Beringian stock of Stamford and Taylor [18] was associated with the Yukon River Basin. In our study, the same Yukon River Basin area is subdivided into three sub-refugia within the range of *Dallia*. Likewise, studies of whitefish (*Coregonus* spp.) largely appear to identify Beringia/Alaska as a refugium, [*e.g*. 12, 13]. Alaskan populations of broad whitefish (*C. nasus*) appear to form a discrete group with the exception of those from the Arctic Coastal Plain, which are more similar to MacKenzie River populations in Northern Canada. This North Beringia and South Beringia divide is apparent in phylogeographic studies of freshwater fishes, but the further refinement of sub-refugia in Beringia in freshwater fishes this paper has not been demonstrated before.

An important possibility is to consider if the apparent genetic structuring of *Dallia* does not represent survival of lineages in separate sub-refugia but rather indicates isolation of lineages into different areas after dispersal from the same refugium. Indirectly, the restricted distribution of *Dallia*, low dispersal ability of the genus and occupation of areas that were nearly entirely unglaciated during the last glacial maximum argue for little post-glacial range expansion. One possible direct approach to address this issue is to estimate the divergence times between interior Alaska and coastal Alaska genetic clusters as populations with the divergence time (*t*) parameter in IM analysis. If the time of divergence between populations were greater than the last glacial maximum, it would indicate that populations predated the last glacial maximum and did not originate from the same refugium. However, the ESS of *t* remained low even the run lengths were increased substantially and IM operating and run parameters were adjusted, indicating poor ability of our dataset to support robust estimates of time of origin. Estimates of intrapopulation coalescent times conducted previously with mitochondrial data for *Dallia* phylogeographic units, which are highly congruent with genetic clusters identified in this study, argue for *Dallia* persisting through glacial cycling in more than one region south of the Brooks Range [19].

The presence of “refugia within refugia” in the Iberian Peninsula are known to confound phylogeographic analyses, leading to invalid conclusions regarding inferred refugia [56]. Consequently, our demonstration of refugia within the Beringian refugium and the potential for genetic structure should be considered in further studies of Beringian phylogeography.

### Patterns of dispersal within Beringia

Estimates of migration rates (individuals per generation) between genetically-delimited populations appear to be mostly congruent with features of the landscape, with variation among migration rates associated to migratory barriers or conduits. Mean migration rate estimates between Coastal Alaska and Arctic Coastal Plain populations of 0.12 to 0.52 individuals a generation indicate restricted movement of individuals around the Brooks Range. This inference is consistent with *a priori* expectations based on large gaps in the distribution of suitable habitat between Arctic coastal plain and Bering Sea drainages. The Yukon and Kuskokwim rivers promote asymmetrical migration in a downstream fashion between the interior Alaska and coastal Alaska populations. The higher rate of downstream migration is compatible with the low dispersal ability expected from *Dallia*.

In the Arctic Coastal Plain to coastal Alaska and the West Beringia to coastal Alaska population comparisons, the IM model produces estimates of migration across what should be impassable barriers for this species. We should consider the results of a general model like IM from a realistic viewpoint. It is very unlikely that *Dallia* are currently moving across the Brooks Range or the Bering Sea, however the migration rates from IM are integrated across time since the populations’ divergence. The estimated migration rates between populations that appear to cross impassable barriers are most likely evidence of historically higher migration around or across the current barriers of the Brooks Range and Bering Sea. Glaciation cycle-mediated changes in sea level and precipitation may be expected to produce changing levels of connectivity between adjacent drainages for populations of *Dallia*, which depend on highly connected coastal plain or wetland hydrological networks to move between adjacent river drainages. Even considering the fact these measures are averages, the very low migration rates of less than one individual per generation present in the Arctic Coastal Plain to coastal Alaska comparison are low enough to allow genetic differentiation under the island model of migration [79].

### Evidence of distribution and population size changes

Paleoclimatic instability affected the distribution and sizes of populations of *Dallia* across Beringia. The fossil record for *Dallia* suggests that prior to the Pleistocene glaciations this genus had a much larger range. Two fossils have been recovered extending the historical range of *Dallia* in both East and West Beringia. In East Beringia, a fossil assigned to *Dallia* was recovered from Homer, Alaska on the Kenai Peninsula from the Late Miocene [80]. The fossil was recovered 400 km from the nearest interior Alaska *Dallia* population and 200 kilometers from the nearest *Dallia* population on the Alaska Peninsula across an ocean strait [81]. In West Beringia, a fossil from the early Middle Pleistocene extends the historical range of the genus 800 km farther west than its current distribution [82]. It is hypothesized that *Dallia* were extirpated from parts of Siberia during the heavy glaciations of the Illinoan [44].

While post-Pleistocene range expansion is typical for many fish species via pro-glacial lake mediated dispersal [83], it appears that the range of *Dallia* was constricted by paleoclimatic instability, from which *Dallia* did not re-expand at the end of the Wisconsinan glaciations. The same pattern of range reduction during the Pleistocene occurred in the Pacific Northwest in *Novumbra*, an ecologically similar relative of *Dallia* [84]. *Novumbra* has one extant species, and while fossil evidence shows the genus was once more widespread in the Pacific Northwest, *Novumbra* currently occurs only in Western Washington State, USA as a result of climatic changes during the Pleistocene [80, 84].

During times of moderate conditions during interglacial periods *Dallia* may have survived in higher numbers and were able to migrate more effectively due to the generally much wetter conditions and presumably increased aquatic connectivity [85]. Changes in climate that reduced precipitation and cooled the climate could have severely impacted abundance and distribution of suitable habitat for *Dallia* in many parts of its range leading to isolation and reduction in population sizes and genetic variability. Sea level retreat on the other hand opened up more habitat along the coasts of Asia and North America. In particular, the Bering land bridge contained large rivers and associated deltas with low gradient and more rainfall than other parts of Beringia [44, 85]. The coastal Alaska genetic cluster of sampled populations was historically part of or very close to the hypothetically suitable *Dallia* habitat of the Bering land bridge and is central in the current distribution of the genus. Therefore, the large estimated *θ* for the coastal Alaska population relative to the other populations suggests that the Bering land bridge and immediately adjacent parts of Alaska in the Yukon-Kuskokwim Delta region historically were and continue to be a center of *Dallia* genetic diversity.

Our analysis supports a much larger ancestral population *θ* than that estimated for contemporary populations, but we were unable to generate reliable estimates of ancestral *θ* in this study for all comparisons. A lack of shared variation between the coastal Alaska and Arctic Coastal Plain populations may prevent convergence of the ancestral *θ* parameter. However, in the case of the coastal Alaska and West Beringia comparison and coastal Alaska and interior Alaska comparison, insufficient data is present to estimate this particular parameter completely.

### How many species of *Dallia* are there?

Two aspects of biological variation among populations of *Dallia* are intriguing. Examined populations of *D. pectoralis* have been found to display spatially segregated karyotypic variation and to have markedly different growth and maturation rates. Karyological data show that sample location 12 (assigned to the coastal Alaska genetic cluster) and sample location 20 (assigned to the Arctic Coastal Plain genetic cluster) originating from *D. pectoralis* sampled within Alaska differ in chromosome number and variability of chromosome number among cells in an individual [57]. The diagnostic morphological characters of Balushkin and Chereshnev [53] used to describe species of *Dallia* in Russia applied to individuals of *D. pectoralis* from sample locations 12 and 20 did not show any significant differences [57].

Further, interior Alaska and coastal Alaska *D. pectoralis* were found to be comprised of distinct mitochondrial lineages with only one shared haplotype in one individual identified [19] and evidence from the nuclear genome examined in this study strongly supports the differentiation of fish from these two areas. Studies of fish from interior Alaska and coastal Alaska geographic areas have demonstrated differences in growth and maturity [49, 50] which may be a result of habit differences and the ability of *Dallia* to adapt to different conditions [48]. The mitochondrial sequence data from the same samples as those used in the karyological study [57] did indicate a high degree of separation between the two studied populations, but the genetic distance observed was well within typical intraspecific distances [19]. In this study, we found isolation of the Arctic Coastal Plain *Dallia* to be quite high with nuclear and combined nuclear and mitochondrial data. Our data indicate private alleles in Arctic Coastal Plain *Dallia* and very low migration rates between the Arctic Coastal Plain and coastal Alaska populations.

The lack of morphological support for the observed karyological differences and the small sample sizes examined by Balushkin and Chereshnev [53] in describing species characteristics points out three major issues with the current status of the taxonomy of *Dallia*. First, morphological variability of *Dallia* across its range is poorly described. Therefore, reported differences may represent within species variability. Second, morphological variation in Arctic fishes can be attributed to rapid adaptation following glacial retreat. Parallel evolution in different phylogenetic lineages can produce convergence on similar ecotypes (i.e. benthic vs limnetic) in short order. Any observed morphological variability could have arisen on a very short (e.g. 20,000 year) time scale and does not necessarily correspond to persistence in glacial refugia over hundreds of thousands or more years. The severity of the Illinoan glaciations also supports that *Dallia* survived only in East Beringia, which would not support separate refugia in Chukotka for the genus at deep time scales [44]. Third, while differences in karyotype are generally indicative of substantial differentiation for vertebrates; again the number of sampled populations is low (n = 2) for these data. Therefore, it is unclear if the karyological data demonstrate variability among populations or evidence of speciation. However, the lack of morphological correspondence with the karyological data suggests that the morphological diagnostic characters put forth so far may not correspond to markers for biological or phylogenetic species boundaries.

An original motivation for this study was to better delineate species of *Dallia* with genetic data. However, our inability to determine nuclear loci sequences from *D. admirabilis* limits our ability to test the hypothesis of a species boundary between *D. admirabilis* and *D. pectoralis*. Regardless, genetic divergence among genetic clusters of *Dallia* evaluated through F_st_ is high in this study, either in STRUCTURE or DAPC defined values. Preliminary microsatellite data indicates that interior Alaska and coastal Alaska samples show significant genetic differentiaion [78]. Under the STRUCTURE model, Arctic Coastal Plain *Dallia* are the most divergent from ancestral allele frequencies, with DAPC Arctic Coastal Plain *Dallia* are not the most distinct from other clusters. It appears that Arctic Coastal Plain *Dallia* represent at the very least a distinct and isolated population of *D. pectoralis*.

Evidence from migration rates indicates that the Bering land bridge served to connect eastern Chukotka with western Alaska. Paleoclimatic instability served to facilitate intercontinental exchange instead of generating species divisions between continents within this fish genus. The populations of *Dallia* examined in this study are similar genetically across the present Bering Sea (West Beringia and coastal Alaska groups of sampling locations). Unfortunately our sampling does not fully encompass the putative species level diversity in the genus. Eastern Chukotka hosts all three nominal species of *Dallia* [53, 54] but our study is confined to one species mostly within Alaska. Therefore we are unable to significantly improve taxonomic understanding for the whole genus.

## Conclusions

Overall, our results support the hypothesis that populations of *Dallia* persisted in Beringia in up to five distinct areas through the most recent glacial oscillations. The pattern of genetic structuring of *Dallia* indicates that (1) the Bering land bridge provided connectivity between Asia and North America for *Dallia* and (2) divergences within Alaska as opposed to across the Bering Sea are greater in this putatively primary freshwater fish. The genetic subdivisions of *Dallia* also indicate that Beringia contains several potential sub-refugia. This demonstration of several sub-refugia within Beringia for a freshwater fish indicates that further phylogeographic studies including Beringia should take steps to avoid being misled by potential diversity within Beringia.

### Availability of supporting data

All nucleotide sequences determined for this study have been submitted to GenBank under accessions KP411389 - KP411458 and KP411459 - KP411534. Previously determined sequences from mitochondrial DNA are available on GenBank as accessions JX961713–JX962051.

## Additional files

Additional file 1:
**Assignment plots from DAPC for K of five populations.**
Additional file 2:
**Figure S2.** Data supporting number of groups of sampled populations (K) from STRUCTURE, InStruct, and DAPC. **Table S2.** Negative log likelihood scores from STRUCTURE analyses and Bayesian Informative Criterion (BIC) from DAPC for different values of K. Additional file 3:
**Figure S3.** Discriminant Analysis of Principal Components (DAPC) scatterplot for K=3 of nuclear intron data. Additional file 4:
**Figure S4.** Posterior distributions from Isolation with Migration (IM) pairiwise comparisons between (A) Coastal Alaska and Interior Alaska, (B) Coastal Alaska and West Beringia and (C) Coastal Alaska and Arctic Coastal Plain populations as dened by DAPC analysis. Additional file 5:
**Supplementary Figure S1.** **Distribution of Alaska blackfish (** ***Dallia pectoralis***) **in Alaska, USA.** Distribution of Alaska blackfish (*Dallia pectoralis*) in Alaska, USA represented as points of known collection localities (red circles, n=412) available in Supplementary Table 1. Additional file 6: Supplementary Table S1. ᅟ Additional file 7: Supplementary Table S3. ᅟ
