# Supplementary material for: Beringian sub-refugia revealed in blackfish (Dallia): implications for understanding the effects of Pleistocene glaciations on Beringian taxa and other Arctic aquatic fauna
Source: BMC Evol Biol. 2015 Jul 19;15:144. doi: 10.1186/s12862-015-0413-2 (PMC4506597; doi:10.1186/s12862-015-0413-2)
Supplement: Additional file 1: — Assignment plots from DAPC for K of five populations. [file 12862_2015_413_MOESM1_ESM.pdf]

Supplementary Document 1: Assignment plots from DAPC for *K* of five populations.

Below a plot of each genetic cluster (1 through 5) generated by DAPC is presented. The particular cluster is on the *x*-axis (1 through 5). Values on the *y*-axis identify particular fish which are associated with GenBank records. The degree of shading indicates the confidence that each particular sample is assigned to a particular genetic cluster. Samples originate from sample locations as as described in the following table:

Sample Location Number	Name	Latittude	Longitude	Number of Samples	Species	Samples
1	Fairbanks	64.8692	-147.8254	6	*D. pectoralis*	2_BP, 4_BP, 5_BP, 6_BP, 7_BP, 10_BP
2	Fairbanks	64.9117	-147.8288	4	*D. pectoralis*	81_GO, 82_GO, 83_GO, 84_GO
4	Kuskokwim Basin	61.1945	-156.1535	4	*D. pectoralis*	99_KU, 100_UKU, 101_UKU, 102_UKU
5	Kuskokwim Basin	61.0812	-156.4840	6	*D. pectoralis*	104_UKU, 105_UKU, 106_UKU, 110_UKU, 111_UKU, 112_UKU
6	Kuskokwim Basin	61.5597	-156.9341	1	*D. pectoralis*	103_UKU
7	Kuskokwim Basin	61.4300	-158.9114	2	*D. pectoralis*	96_KU, 97_KU
8	Kuskokwim Basin	61.5406	-159.3765	1	*D. pectoralis*	98_KU
9	Russian Mission	61.7952	-161.2443	5	*D. pectoralis*	168_RM, 169_RM, 172_RM, 173_RM, 174_RM
10	Togiak	59.0546	-160.3962	3	*D. pectoralis*	12_TO, 13_TO, 14_TO
11	Bethel	60.7904	-161.7799	6	*D. pectoralis*	22_BE, 23_BE, 24_BE, 30_BE, 162_BE, 163_BE
12*	Galena	64.7167	-157.0000	4	*D. pectoralis*	183_GA, 184_GA, 186_GA, 187_GA
14	Nome	64.5061	-165.4305	3	*D. pectoralis*	122_NO, 123_NO, 135_NO
16	North Slope	70.2768	-156.9182	6	*D. pectoralis*	66_NS, 67_NS, 68_NS, 69_NS, 70_NS, 71_NS
17	North Slope	70.1981	-156.1973	2	*D. pectoralis*	72_NS, 73_NS
18	North Slope	70.2528	-155.5849	3	*D. pectoralis*	78_NS ,79_NS, 80_NS
19	North Slope	70.3683	-155.5697	4	*D. pectoralis*	74_NS, 75_NS, 76_NS, 77_NS
20*	Colville River	70.3333	-151.2000	4	*D. pectoralis*	196_CO, 199_CO, 200_CO, 201_CO
21+	Novoe Chaplino	64.4085	-172.2590	9	*D. pectoralis*	136_NC, 137_NC, 138_NC, 139_NC, 141_NC, 142_NC, 143_NC, 144_NC, 145_NC
22+	Ievineem River	65.6808	-172.5542	10	*D. pectoralis*	146_IR, 147_1R, 148_IR, 149_IR, 150_IR, 151_IR, 152_IR, 153_IR, 154_IR, 155_IR

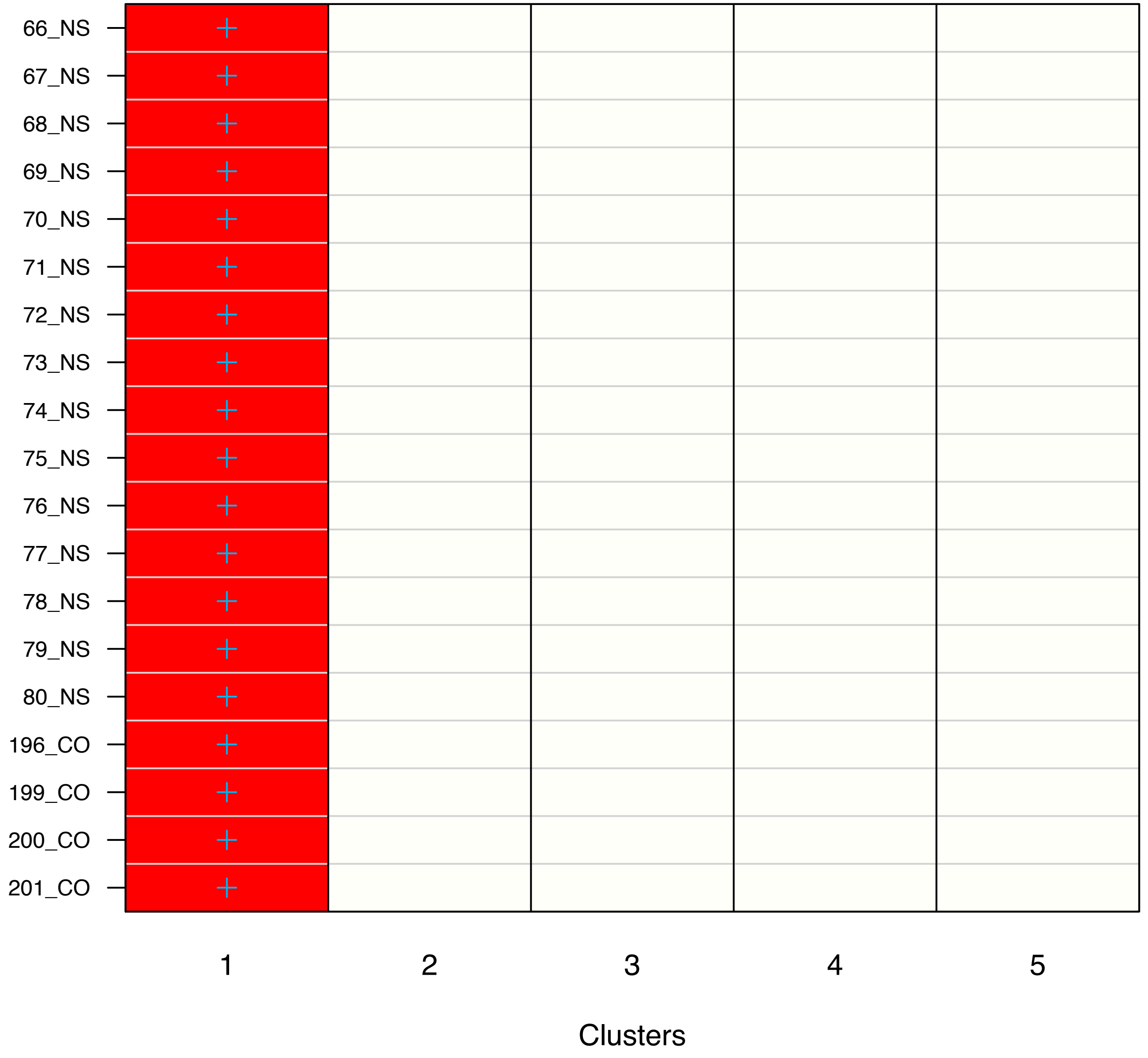
66_NS
67_NS
68_NS
69_NS
70_NS
71_NS
72_NS
73_NS
74_NS
75_NS
76_NS
77_NS
78_NS
79_NS
80_NS
196_CO
199_CO
200_CO
201_CO
1
2
3
4
5
Clusters

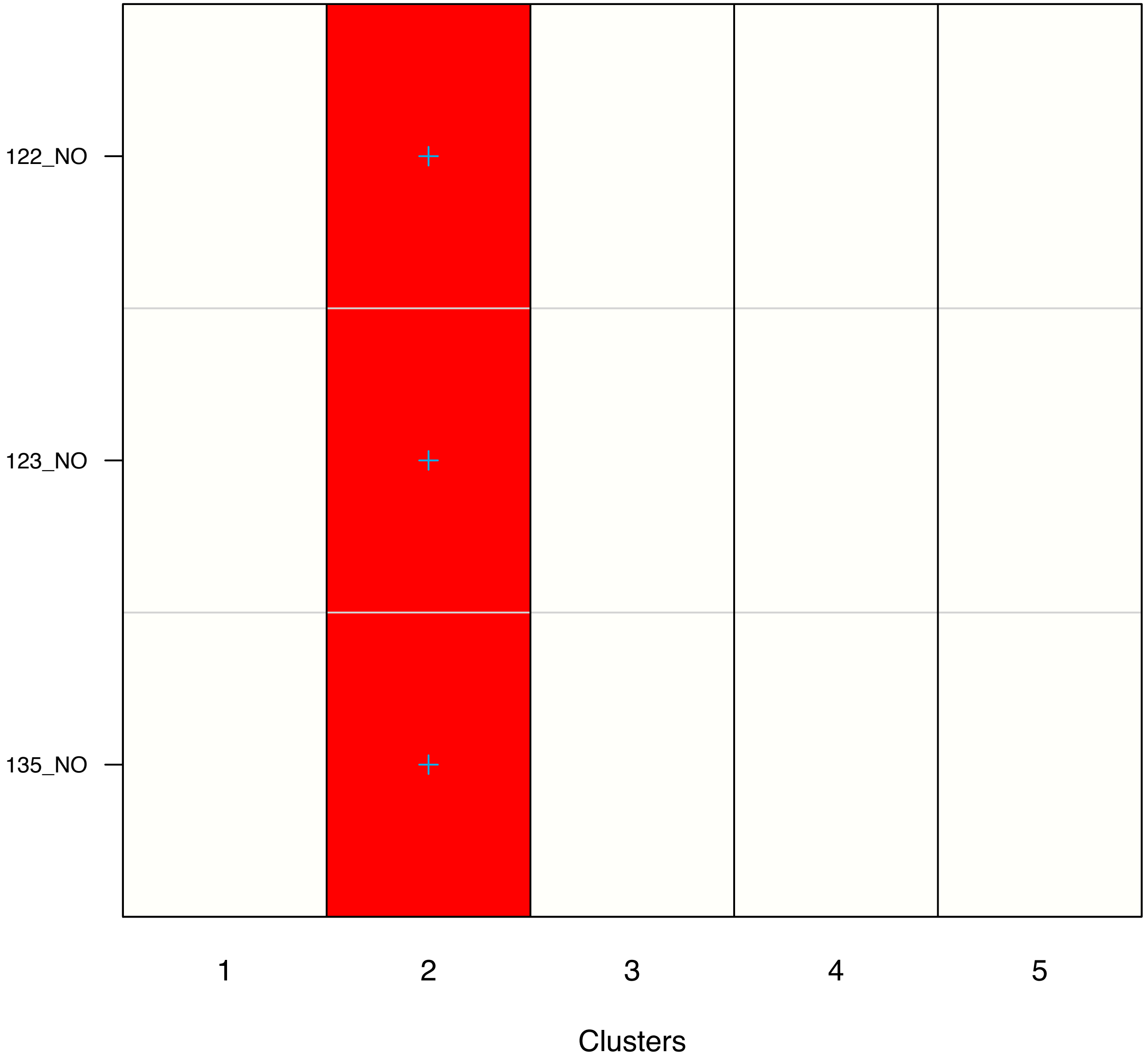
122_NO
123_NO
135_NO
1
2
3
4
5
Clusters

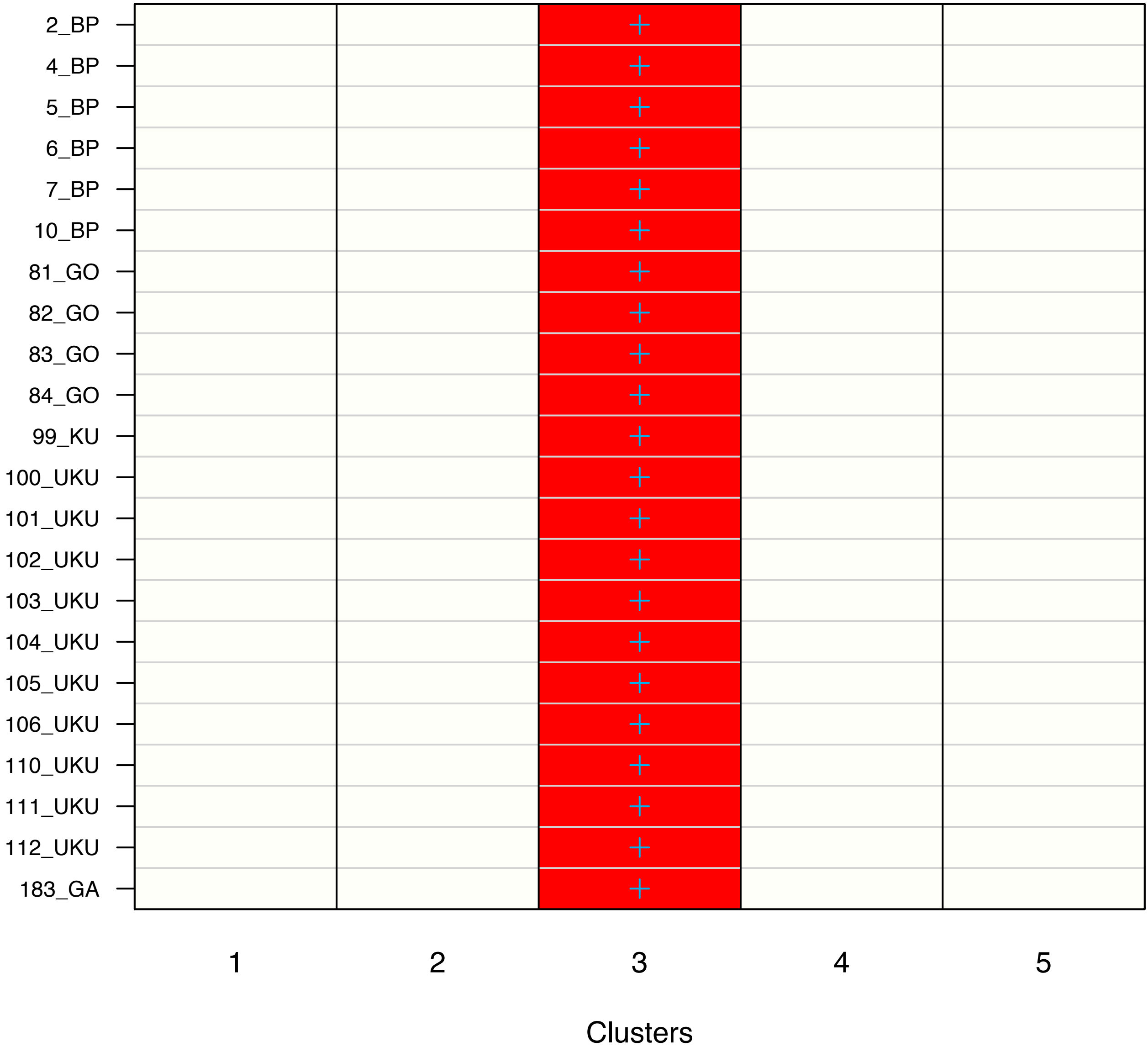
2_BP
4_BP
5_BP
6_BP
7_BP
10_BP
81_GO
82_GO
83_GO
84_GO
99_KU
100_UKU
101_UKU
102_UKU
103_UKU
104_UKU
105_UKU
106_UKU
110_UKU
111_UKU
112_UKU
183_GA
1
2
3
4
5
Clusters

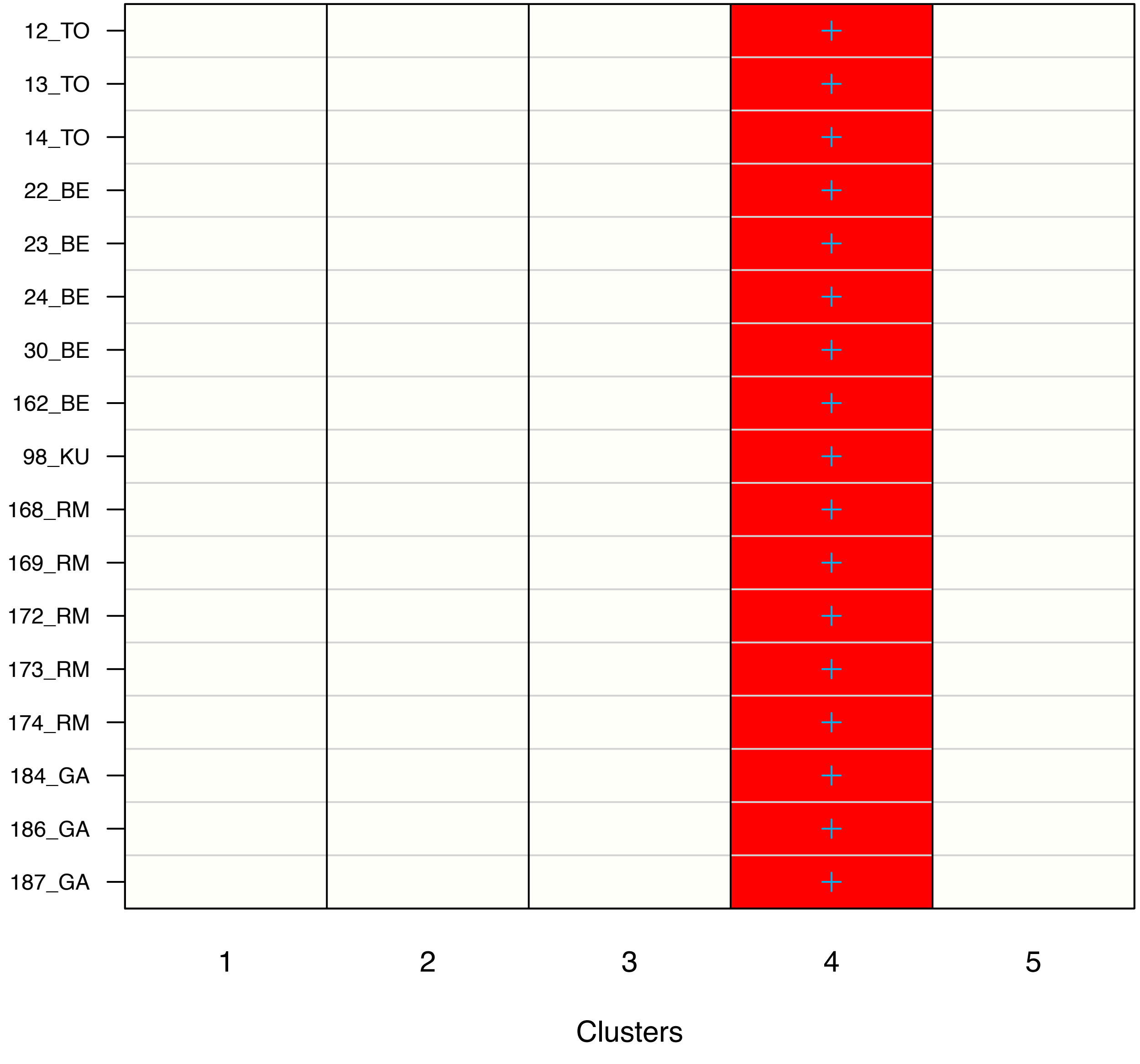
12_TO
13_TO
14_TO
22_BE
23_BE
24_BE
30_BE
162_BE
98_KU
168_RM
169_RM
172_RM
173_RM
174_RM
184_GA
186_GA
187_GA
1
2
3
4
5
Clusters

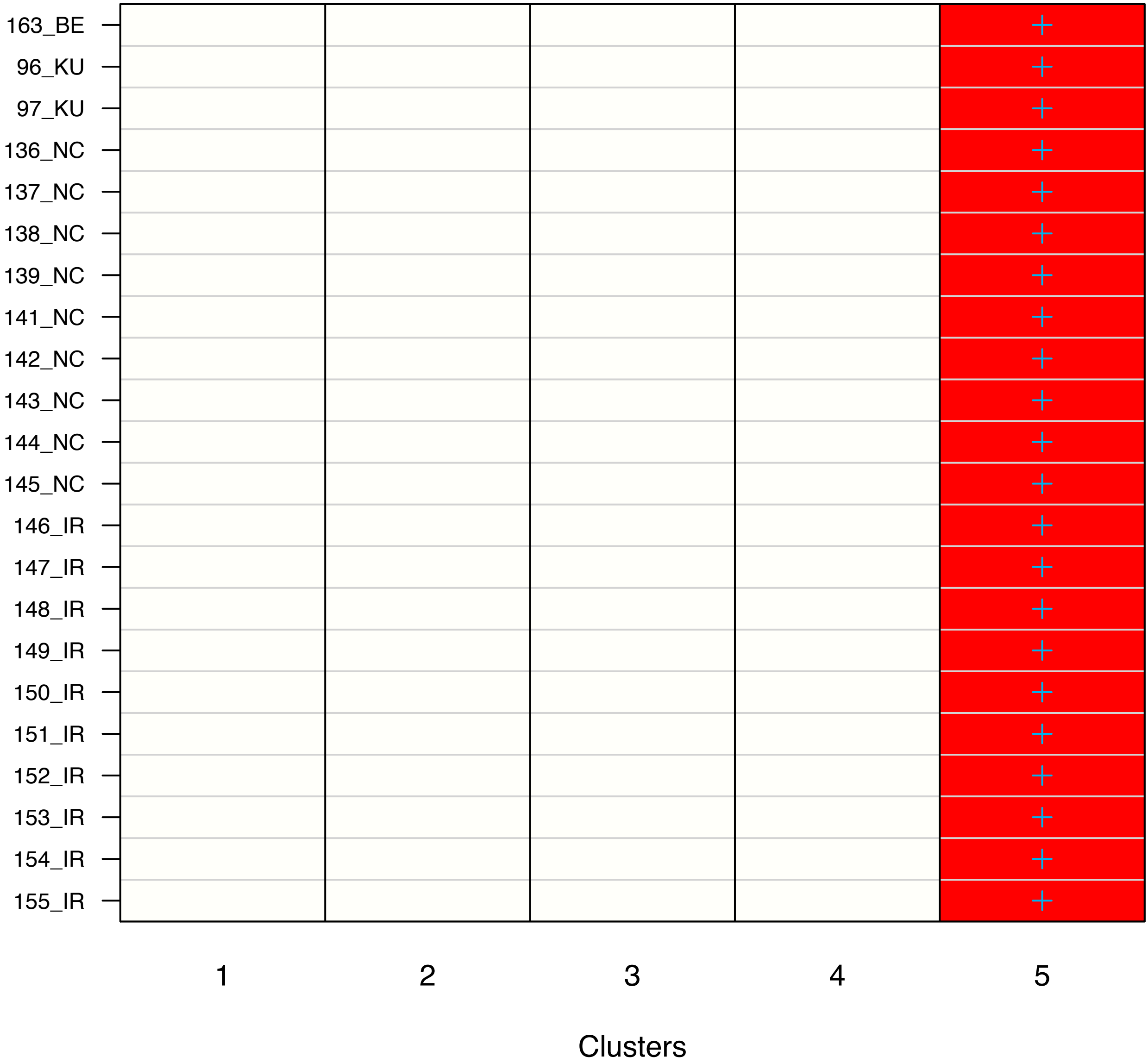

163_BE
96_KU
97_KU
136_NC
137_NC
138_NC
139_NC
141_NC
142_NC
143_NC
144_NC
145_NC
146_IR
147_IR
148_IR
149_IR
150_IR
151_IR
152_IR
153_IR
154_IR
155_IR
1
2
3
4
5
Clusters
