# Supplementary material for: Beringian sub-refugia revealed in blackfish (Dallia): implications for understanding the effects of Pleistocene glaciations on Beringian taxa and other Arctic aquatic fauna
Source: BMC Evol Biol. 2015 Jul 19;15:144. doi: 10.1186/s12862-015-0413-2 (PMC4506597; doi:10.1186/s12862-015-0413-2)
Supplement: Additional file 2: — Figure S2. Data supporting number of groups of sampled populations (K) from STRUCTURE, InStruct, and DAPC. Table S2. Negative log likelihood scores from STRUCTURE analyses and Bayesian Informative Criterion (BIC) from DAPC for different values of K. [file 12862_2015_413_MOESM2_ESM.docx]

##
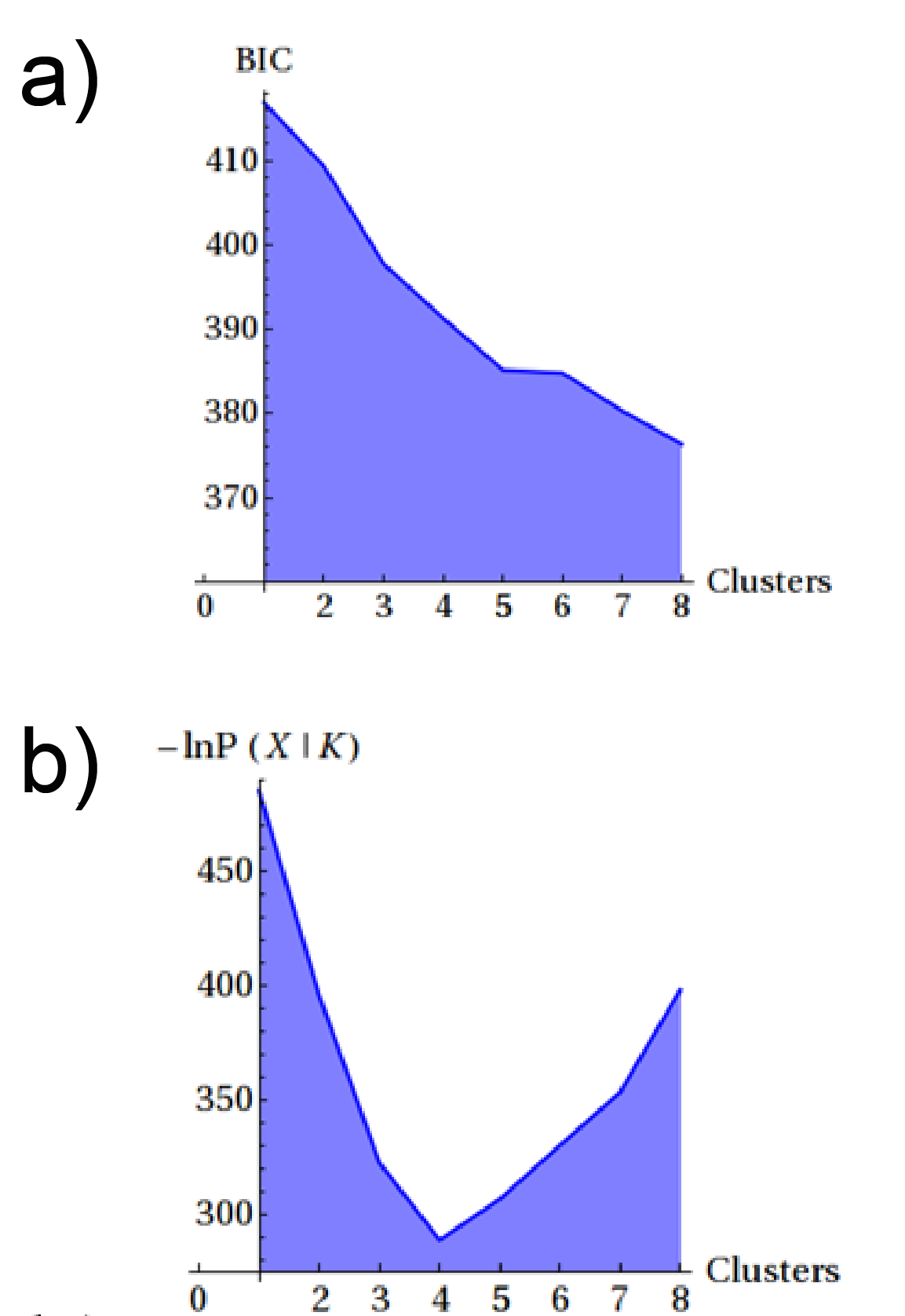


## Supplementary Figure S2 - Data supporting number of groups of sampled populations (*K*) from STRUCTURE, InStruct, and DAPC.

Bayesian Informative Criterion (BIC) or Negative log likelihood (-lnP (X | *K*)) support for the number of groups of sampled populations (*K*) for *K*={2,...,8}. Methods of choosing *K* are shown for (a) DAPC with BIC and (b) STRUCTURE (-lnP (X | *K*)). Values for BIC and -lnP (X | *K*) are presented in Supplementary Table S2 (this document).

## Supplementary Table S2 - Negative log likelihood scores from STRUCTURE analyses and Bayesian Informative Criterion (BIC) from DAPC for different values of *K*.

The Bayesian Informative Criterion (BIC) scores from DAPC for different *K* number of groups are presented and the negative log likelihood (-lnP (X | *K*)) score for different values of *K* groups of sampled populations is presented as averages of two independent STRUCTURE runs.
