# Supplementary material for: Beringian sub-refugia revealed in blackfish (Dallia): implications for understanding the effects of Pleistocene glaciations on Beringian taxa and other Arctic aquatic fauna
Source: BMC Evol Biol. 2015 Jul 19;15:144. doi: 10.1186/s12862-015-0413-2 (PMC4506597; doi:10.1186/s12862-015-0413-2)
Supplement: Additional file 3: — Figure S3. Discriminant Analysis of Principal Components (DAPC) scatterplot for K=3 of nuclear intron data. [file 12862_2015_413_MOESM3_ESM.docx]

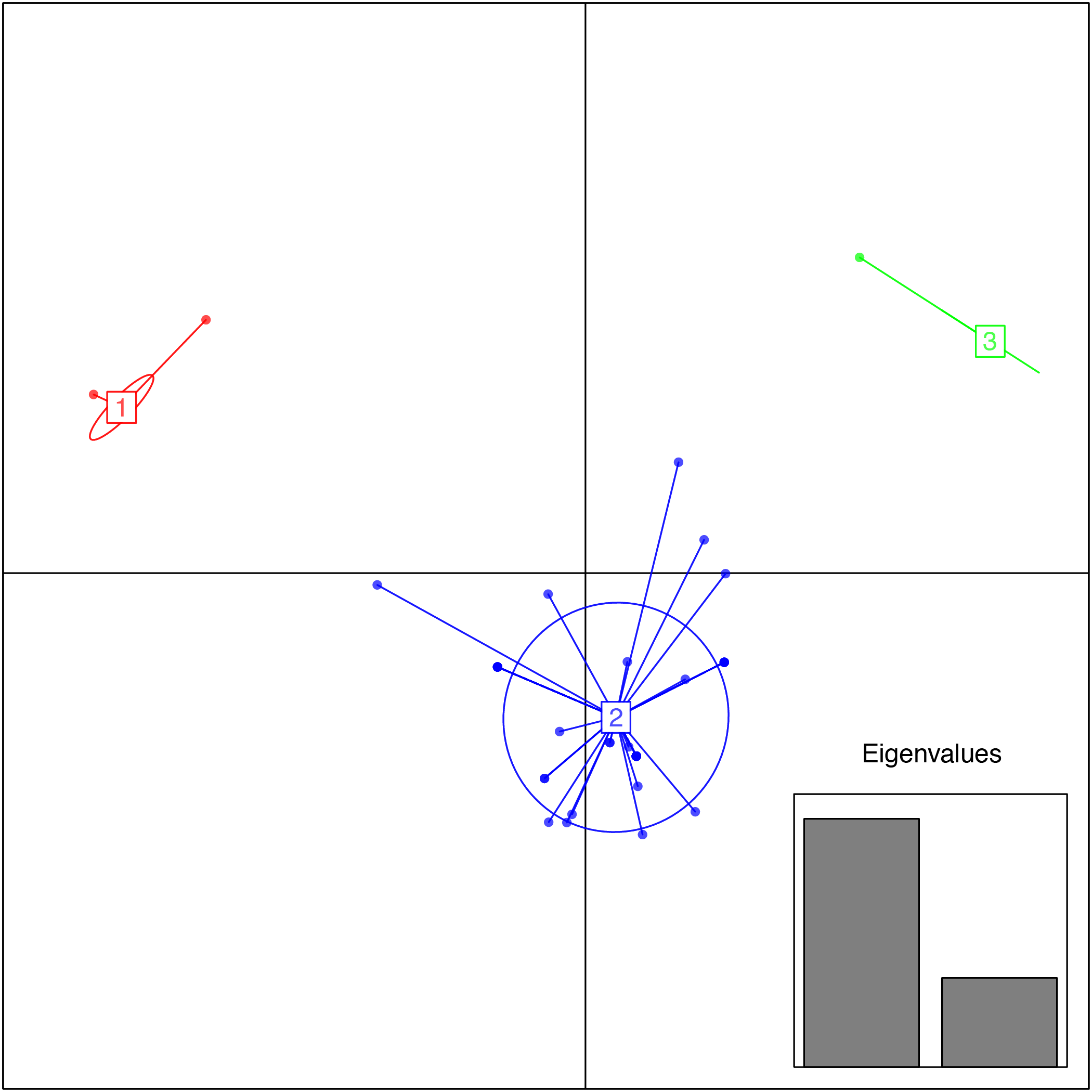


**Supplementary Figure S3: Discriminant Analysis of Principal Components (DAPC) scatterplot for *K*=3 of nuclear intron data**

Discriminant Analysis of Principal Components (DAPC) scatterplot for *K=*3, depicting the clustering of individuals based on K-means clustering of principal components. Eigenvalues representing between to within group variation for linear combinations of principal components are included in this figure. Cluster 1 consists of eighteen individuals from the Arctic Coastal Plain (sample locations 16-20, n=17) and one individual from sample location 12 (n=1). Cluster 2 contains forty-eight individuals, most (n=42) from Western Coastal Alaska and Chukotka Alaska (sample location 7 [n=2], sample location 8 [n=1], sample location 9 [n=5], sample location 10 [n=3], sample location 11 [n=6], sample location 12 [n=3], sample location 14 [n=3]; sample location 21 [n=10], sample location 22 [n=9]) with six individuals placed in this group from other geographic regions (Arctic Coastal Plain sample location 16 [n=1] and sample location 20 [n=1]; Interior Alaska sample location 1 [n=2], sample location 4 [n=1], sample location 5 [n=1]). Bayesian Informative Criterion (BIC) output from DAPC analysis and assignment plots for each cluster are presented below in this document.


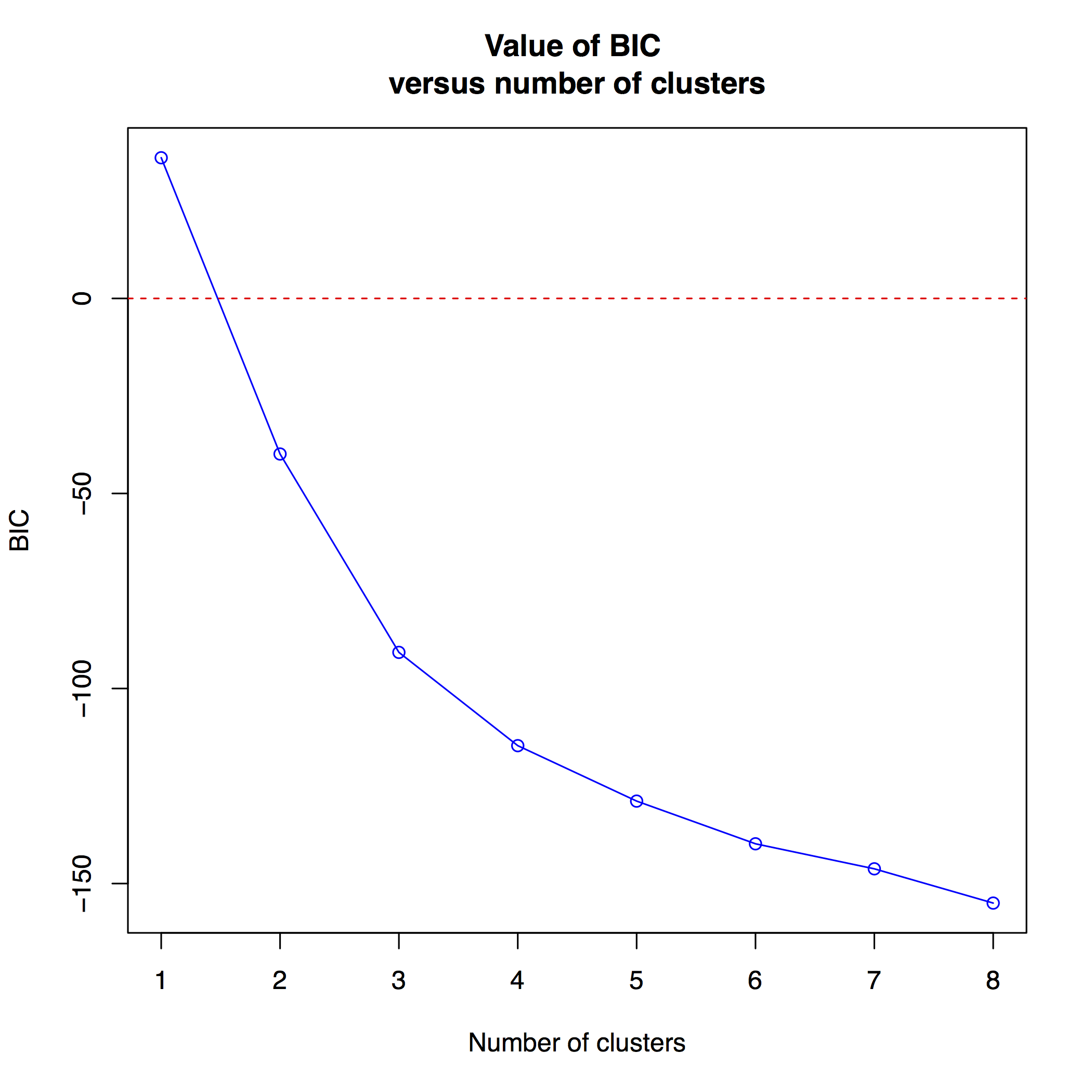


**Supplementary Document 2: BIC plot versus number of clusters.**


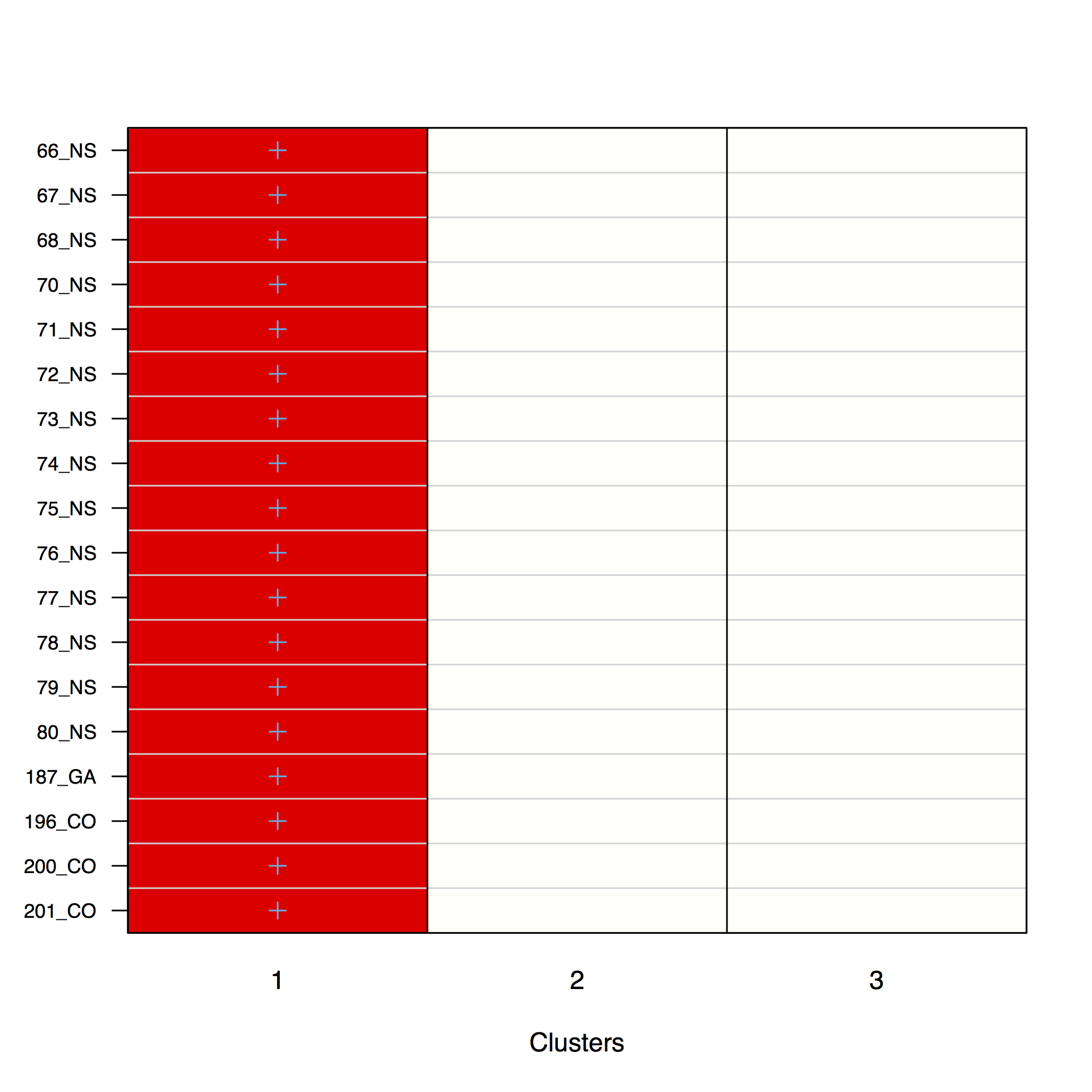


**Supplementary Document 2: Assignment plot for Cluster 1 from nuclear data.**

See Supplementary Document 1 to relate sample names to sample locations directly.


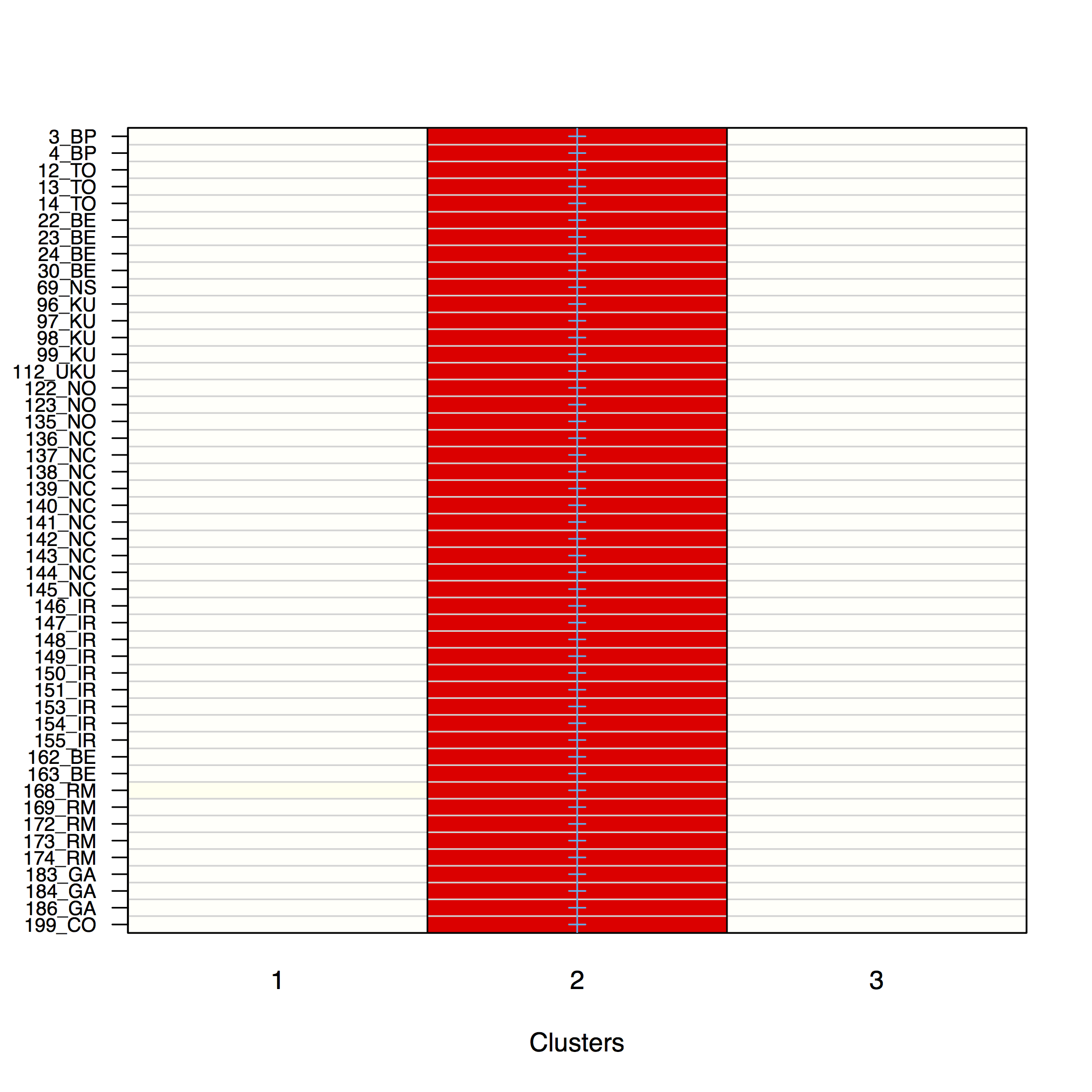


**Supplementary Document 2: Assignment plot for Cluster 2 from nuclear data.**

See Supplementary Document 1 to relate sample names to sample locations directly.


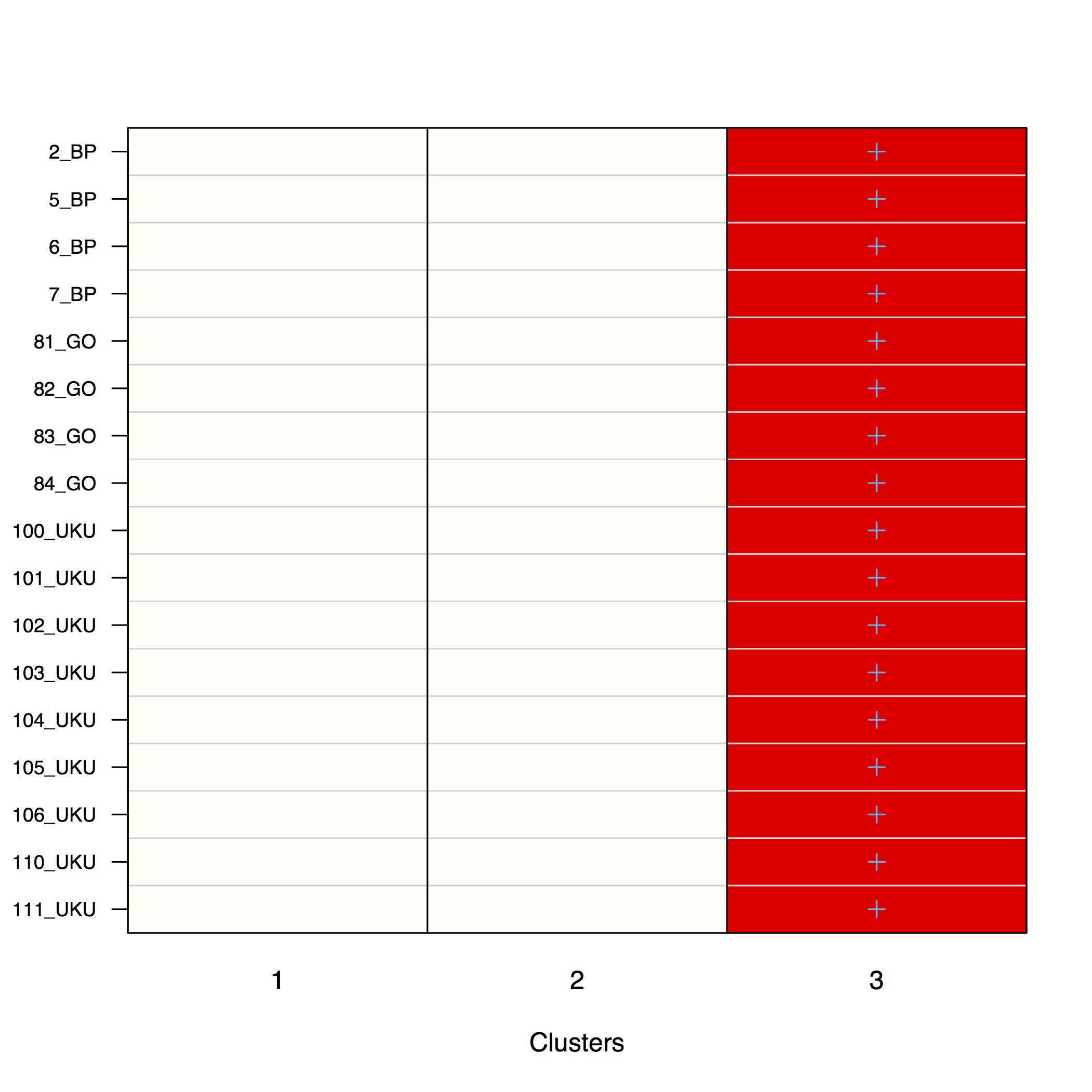


**Supplementary Document 2: Assignment plot for Cluster 3 from nuclear data.**

See Supplementary Document 1 to relate sample names to sample locations directly.
