# Supplementary material for: Beringian sub-refugia revealed in blackfish (Dallia): implications for understanding the effects of Pleistocene glaciations on Beringian taxa and other Arctic aquatic fauna
Source: BMC Evol Biol. 2015 Jul 19;15:144. doi: 10.1186/s12862-015-0413-2 (PMC4506597; doi:10.1186/s12862-015-0413-2)

Supplemental Figure S4 - Posterior distributions from Isolation with Migration (IM) pairwise comparisons between (A) Coastal Alaska and Interior Alaska, (B) Coastal Alaska and West Beringia and (C) Coastal Alaska and Arctic Coastal Plain populations as defined by DAPC analysis.

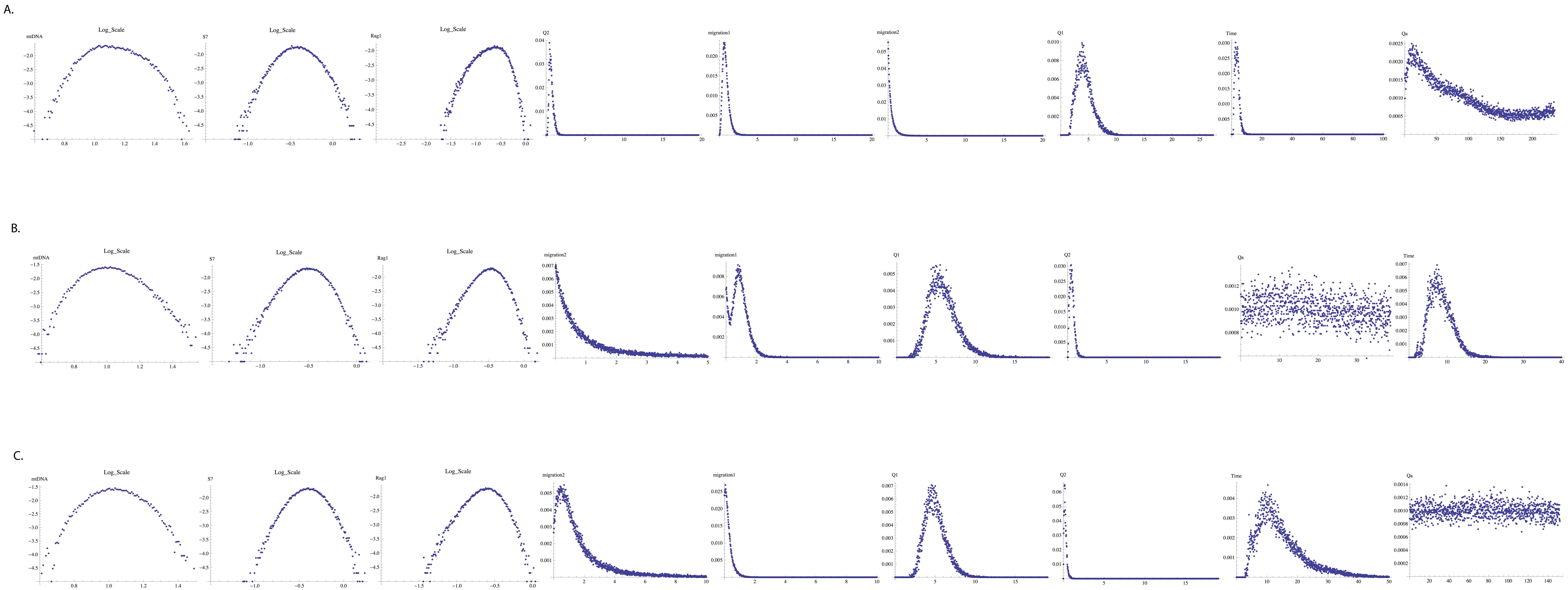

Supplement: Additional file 4: — Figure S4. Posterior distributions from Isolation with Migration (IM) pairiwise comparisons between (A) Coastal Alaska and Interior Alaska, (B) Coastal Alaska and West Beringia and (C) Coastal Alaska and Arctic Coastal Plain populations as dened by DAPC analysis. [file 12862_2015_413_MOESM4_ESM.pdf]
