# Supplementary material for: Beringian sub-refugia revealed in blackfish (Dallia): implications for understanding the effects of Pleistocene glaciations on Beringian taxa and other Arctic aquatic fauna
Source: BMC Evol Biol. 2015 Jul 19;15:144. doi: 10.1186/s12862-015-0413-2 (PMC4506597; doi:10.1186/s12862-015-0413-2)
Supplement: Additional file 5: — Supplementary Figure S1. Distribution of Alaska blackfish ( Dallia pectoralis) in Alaska, USA. Distribution of Alaska blackfish (Dallia pectoralis) in Alaska, USA represented as points of known collection localities (red circles, n=412) available in Supplementary Table 1. [file 12862_2015_413_MOESM5_ESM.docx]

##
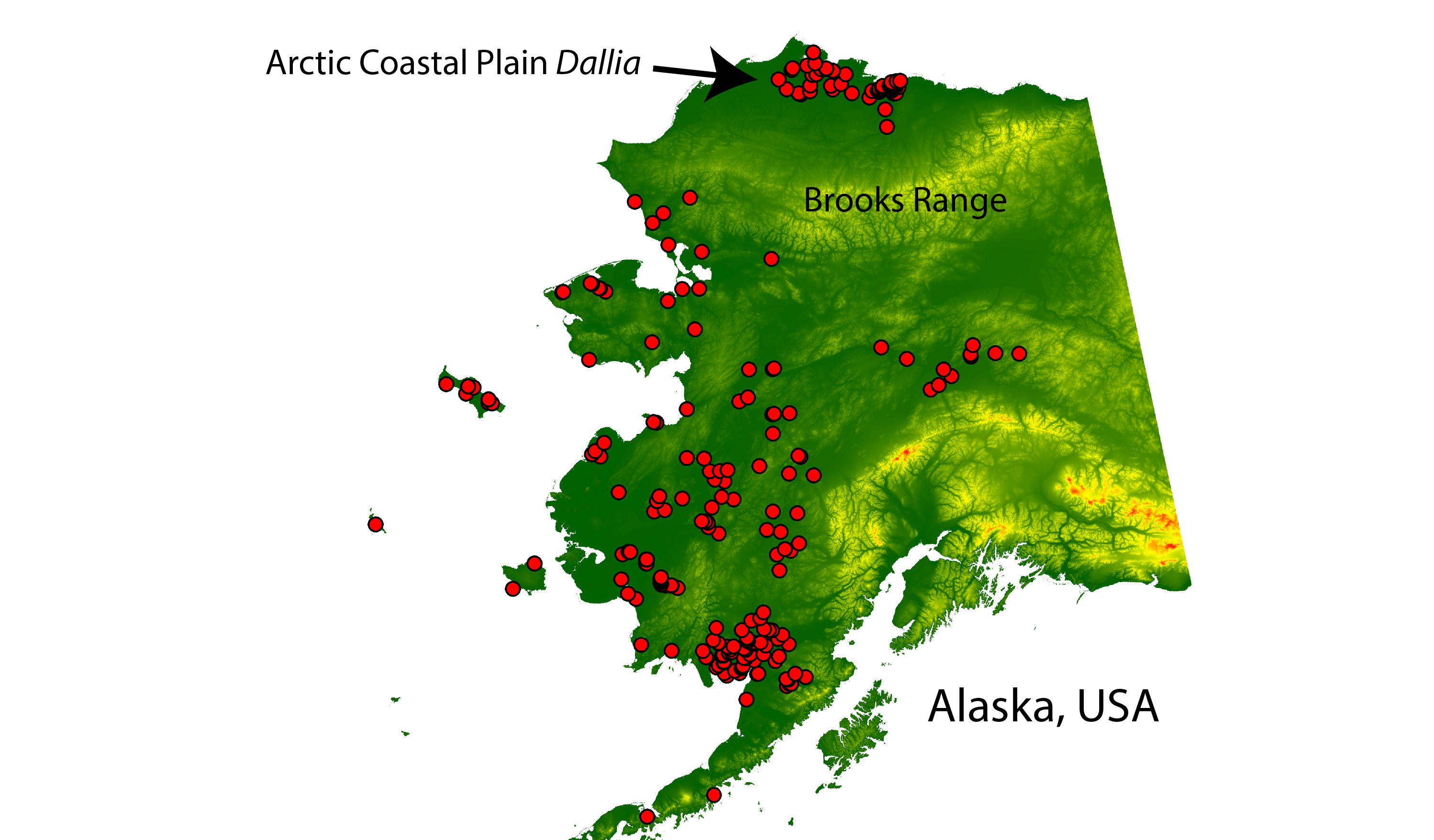


## Supplmentary Figure S1 - Distribution of Alaska blackfish (*Dallia pectoralis*) in Alaska, USA

Distribution of Alaska blackfish (*Dallia pectoralis*) in Alaska, USA represented as points of known collection localities (red circles, n=412) available in Supplementary Table 1. Collections are shown on top of a Digital Elevation Model created by the United States Geological Survey color coded by elevation from green to yellow (low to high). Collection locations were documented by the authors, Alaska Department of Fish and Game, Dr. Lawrence Moulton (MJM Research), or were compiled by the Alaska Natural History Project from existing literature. The distribution of Alaska blackfish appears to be comprised of two major divisions on mainland Alaska, with fish of the Arctic Coastal Plain apparently separated from other Alaska blackfish.
